# High Rate Triggers Increased Atrial Release of BMP10, A Biomarker for Atrial Fibrillation and Stroke, and BMP10 Affects Ventricular Cardiomyocytes

**DOI:** 10.1161/CIRCEP.125.013834

**Published:** 2025-10-15

**Authors:** Laura C. Sommerfeld, Jessica Schrapers, Karl-Felix Müller, Laura Bravo-Merodio, Bente Siebels, A. M. Stella Vermeer-Stoter, Bangfen Pan, Grit Höppner, Christopher O’Shea, Julius Ridder, Hartwig Wieboldt, Paulina Sander, Tanja Zeller, Winnie Chua, Yanish J.V. Purmah, Robert S. Gardner, Nathan R. Tucker, Paulus Kirchhof, Marc N. Hirt, Thomas Eschenhagen, Justus Stenzig, Larissa Fabritz

**Affiliations:** University Center of Cardiovascular Science (UCCS), University Medical Center Hamburg-Eppendorf, UKE (L.C.S., J.R., H.W., P. S, T.Z., M.N.H., L.F.).; Department of Cardiology, University Heart & Vascular Center Hamburg (L.C.S., J. R., H. W., P.K., L.F.).; DZHK (German Center for Cardiovascular Research), partner site Hamburg/Kiel/Lübeck (L.C.S., J. Schrapers, K.-F.M., A.M.S.V.-S., B.P., J. R., P. S., T.Z., P.K., M.N.H., T.E., J. Stenzig, L.F.).; Institute of Experimental Pharmacology & Toxicology, UKE, Hamburg, Germany (J. Schrapers, K.-F.M., A.M.S.V.-S., B.P., G.H., M.N.H., T.E., J. Stenzig).; Cancer & Genomic Sciences, University of Birmingham, United Kingdom (L.B.).; Cardiovascular Sciences, University of Birmingham, United Kingdom (L.B.-M., C.O.S., W.C., Y.J.V.P., P.K., L.F.).; Center of Diagnostics, Section Mass Spectrometry & Proteomics/Core Facility Mass Spectrometric Proteomics, UKE, Hamburg, Germany (B.S.).; Division of Biomedical Sciences, Warwick Medical School, Clinical Sciences Research Laboratory, Coventry (C.O.S.).; Sandwell & West Birmingham Hospitals NHS Trust, Smethwick, United Kingdom (Y.J.V.P.).; Department of Pharmacology, SUNY Upstate Medical University, Syracuse, NY (R.S.G., N.R.T.).

**Keywords:** atrial fibrillation, bone morphogenetic proteins, cardiac pacing, artificial, cardiomyopathies, heart failure, optogenetics, tachycardia-induced cardiomyopathy, tissue engineering

## Abstract

**BACKGROUND:**

BMP10 (bone morphogenetic protein 10) is a ligand of the TGF (transforming growth factor) β superfamily secreted mainly by atrial cardiomyocytes. Elevated BMP10 blood concentrations predict atrial fibrillation (AF), AF recurrence after ablation, and AF-related cardiovascular complications like stroke. The conditions increasing BMP10 secretion and the downstream effects of BMP10 in cardiomyocytes are poorly understood. We assessed BMP10 secretion dynamics and BMP10 effects in a human 3-dimensional model of atrial and ventricular engineered heart tissue (EHT).

**METHODS:**

Cardiomyocytes (atrial and ventricular) differentiated from human induced pluripotent stem cells were cast into a fibrin-matrix to generate EHT. Atrial EHTs were optogenetically paced (3–5 Hz) or maintained at intrinsic beating rate for 24 hours up to 15 days. Release of BMP10 and other cardiac biomarkers from EHT was quantified. BMP10 plasma concentrations were compared between 1370 patients with different atrial rhythms at blood draw. Additionally, ventricular EHTs were exposed to BMP10 for 10 days.

**RESULTS:**

Atrial but not ventricular EHT released BMP10 within 48 hours of culture. High-rate optogenetic pacing increased atrial EHT BMP10 release by ≈3-fold after a latency of at least 24 hours post initiation of pacing. BMP10 plasma concentrations were elevated in patients with documented AF compared with sinus rhythm and even higher in patients with current AF. BMP10 induced upregulation of TGFβ pathway transcripts, increased expression of genes related to AF and heart failure, including *PITX2* and *NPPB*, and increased relative contraction times in ventricular EHTs.

**CONCLUSIONS:**

High atrial rates elevate BMP10 expression and release, and higher plasma concentrations of BMP10 are observed in patients with active AF. BMP10 exposure induces transcriptomic changes linked to AF and heart failure in ventricular EHT. These findings support BMP10 as a biomarker and potential mediator of AF-related remodeling and tachycardiomyopathy.

**WHAT IS KNOWN?**

- BMP10 (bone morphogenetic protein 10) is a secreted member of the TGF (transforming growth factor)-β-superfamily expressed in the heart.
- Elevated blood concentrations of BMP10 are associated with atrial fibrillation and its complications.

**WHAT THE STUDY ADDS**

- High atrial rates lead to BMP10 release from engineered atrial cardiac tissue.
- Atrial cardiomyocyte-derived, released BMP10 activates BMP signaling in cardiac fibroblasts.
- BMP10 activates cardiac expression of genes associated with atrial fibrillation and heart failure, including *PITX2* and *NPPB*, in human ventricular cardiomyocytes.
- BMP10 may play a role in atrial cardiomyopathy and tachyarrhythmia-induced cardiomyopathy.

Atrial fibrillation (AF) affects 60 million individuals worldwide.^1^ Many patients remain undiagnosed.^2^ Early initiation of rhythm-control therapy can prevent AF-related complications, including cardiovascular death, stroke, and heart failure hospitalizations^3^ and is recommended in current guidelines.^4,5^ Quantifiable markers of AF burden would simplify effective early rhythm control.

Recently, BMP10 (bone morphogenetic protein 10) was identified as a putative blood biomarker for AF. Elevated blood concentrations of BMP10 were shown to be associated with AF,^6^ stroke in patients with AF,^7^ recurrent AF after ablation,^8,9^ and a lower chance of attaining sinus rhythm at follow-up.^10^ BMP10 in combination with other biomarkers can detect patients with prevalent AF^6^ and can identify patients with AF at high risk of cardiovascular events.^11^ Understanding the conditions leading to BMP10 release from the atria and the functional effects of BMP10 in the heart could help to better define the clinical use of BMP10.

BMP10 is a secreted protein, detectable in blood, and almost exclusively produced by the heart with minimal contribution from the liver. BMP10 is expressed during mammalian embryogenesis and affects cardiomyocyte development and growth.^12^ In the adult heart, *BMP10* gene expression is restricted to the atria.^8^

Despite its implication as an AF-associated biomarker, triggers for increased BMP10 expression and subsequent release are not known. Cardiac signaling resulting from BMP10 exposure in adulthood and its possible contribution to AF pathogenesis have not been studied. We investigated BMP10 release and downstream signaling in human cardiomyocytes using atrial and ventricular engineered heart tissue (EHT; aEHT/vEHT) as human in vitro models.^13,14^

## Methods

Anonymized data that support the findings of this study are available from the corresponding author on reasonable request. Detailed methods can be found in the Supplemental Material.

### EHT Generation, Culture, and Contractility Analysis (aEHT/vEHT)

EHTs were generated and assessed using established protocols.^13,15^ See Supplemental Material.

### Optogenetic Fast Pacing of aEHT

To enable longer-term optogenetic pacing (3–5 Hz), the light-sensitive nonselective cation channel CheRiff2.0 was used. Atrial EHTs were transduced during casting with an AAV6 (adeno-associated virus type 6) conferring channel expression under the control of a cardiomyocyte-specific cTNT promoter. LEDs were addressed to rhythmically elicit blue light pulses (465 nm, 0.12 mW/mm^2^, 45 ms stimuli).

### Protein Concentration Analysis in EHT Media

BMP10 and ANP (atrial natriuretic peptide) in culture media were quantified by ELISA according to the manufacturer’s instructions (EK18108; Human BMP-10 ELISA kit PicoKine, Boster, Pleasanton; EIAANP; ANP Competitive ELISA Kit, Thermo Fisher Scientific, Carlsbad). Fresh EHT culture media were confirmed not to contain any detectable BMP10. hsTnI (high sensitive troponin I), NT-proBNP (N-terminal pro-B-type natriuretic peptide), and glucose media concentrations were measured using Abbott Architect Assays on Architect i2000 and c8000 Immuno Assay Analyzers (Abbott Diagnostics).

### Fibroblast Exposure to Conditioned Culture Media of aEHT

To assess the biological activity of aEHT-secreted BMP10, culture media from aEHT were transferred to isogenic hiPSC-derived quiescent cardiac fibroblasts, a cell type known to express BMP10 receptors. Quiescent cardiac fibroblast differentiation was performed as previously described^16^ with adaptations. Fibroblasts were exposed to conditioned media collected from (1) unpaced aEHT, (2) fast-paced aEHT (4 Hz, 18 days), (3) 10 ng/mL rhBMP10 (recombinant human BMP10), or (4) vehicle control for 24 hours. Media were left on aEHT for 48 hours before transfer. To prevent activation of fibroblasts by horse serum contained in the aEHT culture medium, the TGF (transforming growth factor)-β inhibitor SB-431542 was used (5 µmol/L). Following exposure, fibroblast lysates were probed for SMAD1/5/9 phosphorylation (directly reflecting BMP10 receptor activation) by Western blot.

### BMP10 Concentration Analysis in Patient Plasma

Plasma BMP10 concentrations in patients were determined from the Birmingham and Black Country Atrial Fibrillation Registry, which recruited consecutive patients from the Sandwell and West Birmingham NHS (National Health Service) Trust (United Kingdom) between September 2014 and February 2018, as reported.^6,17^ Patients either had confirmed AF or other cardiovascular conditions (without AF) based on the CHA₂DS₂-VASc score; those without prior AF underwent 7-day ECG monitoring. The study was ethically approved (Integrated Research Application System IRAS ID 97753) and complied with the Declaration of Helsinki. All participants provided written informed consent. Blood samples were immediately processed, stored at −80 °C, and analyzed centrally as reported before, using a high-throughput, high-precision assay (Roche Diagnostics, Penzberg, Germany).^6,17^ For clinical characteristics of patients, see Table S1.

### Protein Analysis by Western Blot

Antibodies were directed against SMAD1 (1:1000 in 5% milk/Tris-Buffered Saline with Tween 20, CST no. 9743), phospho-SMAD1 (pSer463/465)/SMAD5 (Ser463/465)/SMAD-9 (Ser465/467, 1:1000 in 5% milk/Tris-Buffered Saline with Tween 20, CST no. 13820), and GAPDH as loading control (1:2000 in 5% milk/Tris-Buffered Saline with Tween 20, HyTest no. 564).

### BMP10 Exposure of vEHT

Effects of BMP10 on vEHT contractility were assessed by both acute (30 min) and longer-term (10 days) exposure of vEHT to recombinant human BMP10 (rhBMP10; 2926-BP-025, R&D Systems, Minneapolis) dissolved in water containing 0.1% BSA and 4 mmol/L HCl (vehicle).

For acute experiments, vEHTs were exposed to accumulating concentrations of rhBMP10 for 30 minutes each. We aimed for BMP10 concentrations comparable to those in the heart-lung circulation, which we estimated to be 2 to 20× higher than peripheral vein concentrations observed in patients.

### Expression Analysis

Gene expression was analyzed by reverse transcription quantitative polymerase chain reaction, and RNA sequencing and mass spectrometry-based proteomic analysis of EHTs was performed. The RNA sequencing data generated in this study have been deposited in the NCBI Gene Expression Omnibus (GEO) under accession number GSE306533.

### Statistical Methods

For statistical analysis not related to RNA sequencing or mass spectrometry, GraphPad Prism software (v.10.0; Dotmatics, Boston) was used. Statistical tests were applied as appropriate and are indicated in figure legends. Sample numbers refer to n/n (number of EHTs/batches) and graphs depict individual values and mean±SEM unless stated otherwise.

## Results

### BMP10 Expression and Secretion Is Restricted to Atrial EHT, Increases With High Rate Pacing, and Activates BMP Signaling in Cardiac Fibroblasts

**Figure 1.**
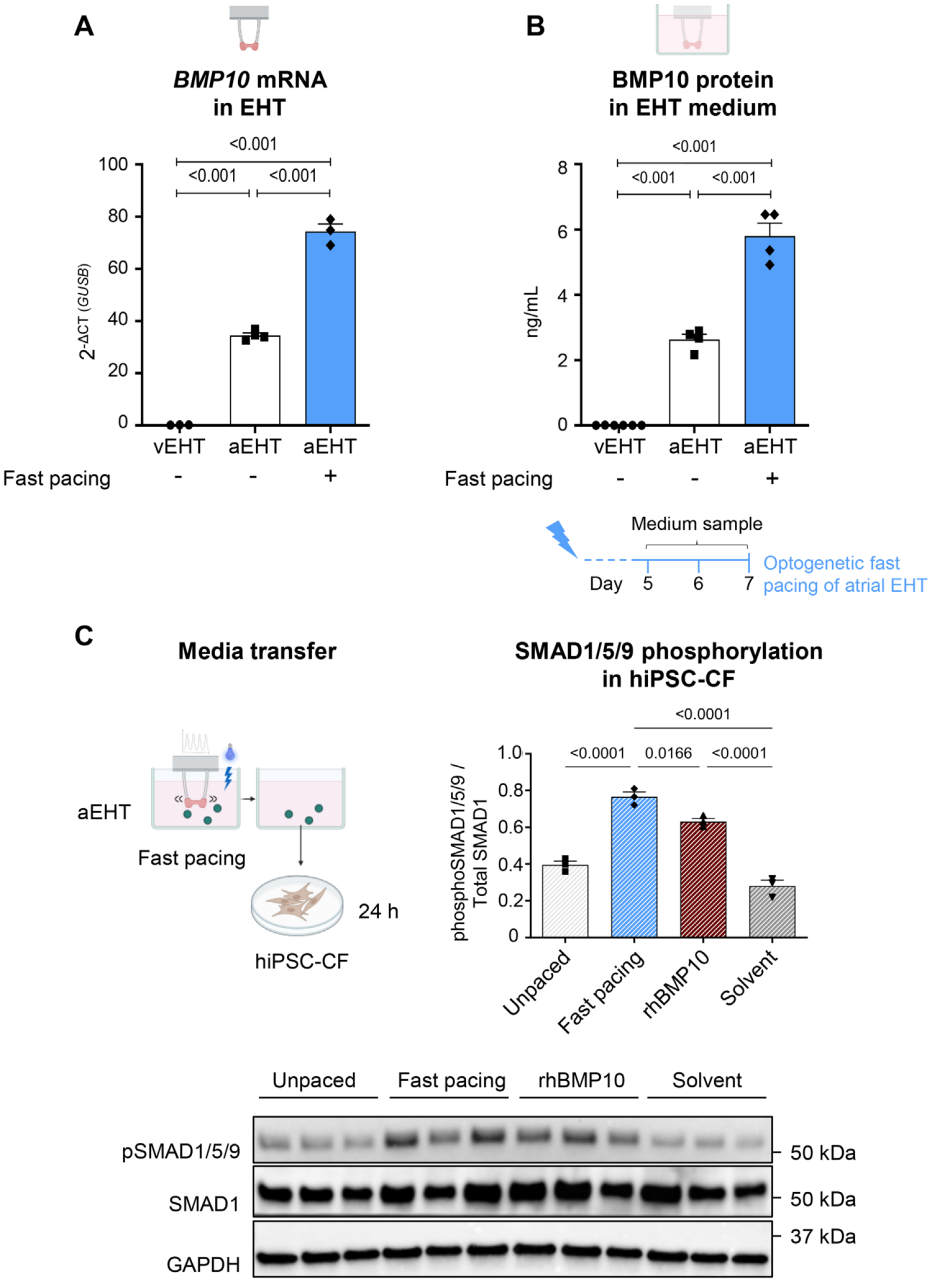
**BMP10 (bone morphogenetic protein 10) expression and release by engineered heart tissue (EHT) and conditioned media effect on cardiac fibroblasts. A**, *BMP10* mRNA abundance from spontaneously beating ventricular EHT (vEHT), spontaneously beating aEHT, and atrial EHT (aEHT) paced at 3 Hz for 7 days (+, blue, filled bars; quantitative polymerase chain reaction, n=3–4/1). **B**, BMP10 in media from aEHT as in **A**, collected after 48 h of aEHT culture (day 5–7, ELISA, n=4–6/1). **C**, Human induced pluripotent stem cell-derived quiescent cardiac fibroblasts (hiPSC-CF) were exposed to media transferred from aEHT left at intrinsic beating frequency (unpaced), optogenetically fast-paced at 4 Hz for 18 days, or to media containing either 10 ng/mL rhBMP10 (recombinant human BMP10) or its solvent only, for 24 h. Quantification of SMAD1/5/9 phosphorylation (phosphoSMAD1/5/9) from Western blot experiments (normalized to total SMAD1 protein expression, n=3 wells/group). One-way ANOVA followed by Tukey multiple comparisons test, *P*_adj_ reported for all comparisons.

*BMP10* mRNA was detectable in both aEHT and vEHT, although at ≈200-fold higher abundance in aEHT compared with vEHT (Figure 1A).

Tachypacing of aEHT, an established model to study AF,^18,19^ at 3 Hz, increased *BMP10* mRNA expression in aEHT by 2-fold (Figure 1A). Average BMP10 concentrations in culture medium of aEHT were ≈2.6 ng/mL after 48 hours in culture. In contrast, BMP10 was hardly detectable in vEHT media (Figure 1B). Atrial EHT pacing led to a 2-fold increase in BMP10 release (≈5.8 ng/mL within 48 hours) compared with unpaced controls (Figure 1B).

To evaluate the biological activity of BMP10 secreted by aEHT, hiPSC-derived quiescent cardiac fibroblasts were treated with conditioned media collected from aEHT. SMAD1/5/9 phosphorylation, reflecting BMP signaling activity, was higher in cells exposed to media from fast-paced aEHT compared with those treated with control media (Figure 1C). SMAD1/5/9 phosphorylation was also elevated following treatment with 10 ng/mL rhBMP10, a concentration that mirrors the BMP10 concentrations accumulated in aEHT media after fast pacing (Figure 1C). *ID1* and *SMAD9* gene expression was similarly increased (Figure S1).

### BMP10 Secretion Increases With Fast Pacing, But With a Delay

**Figure 2.**
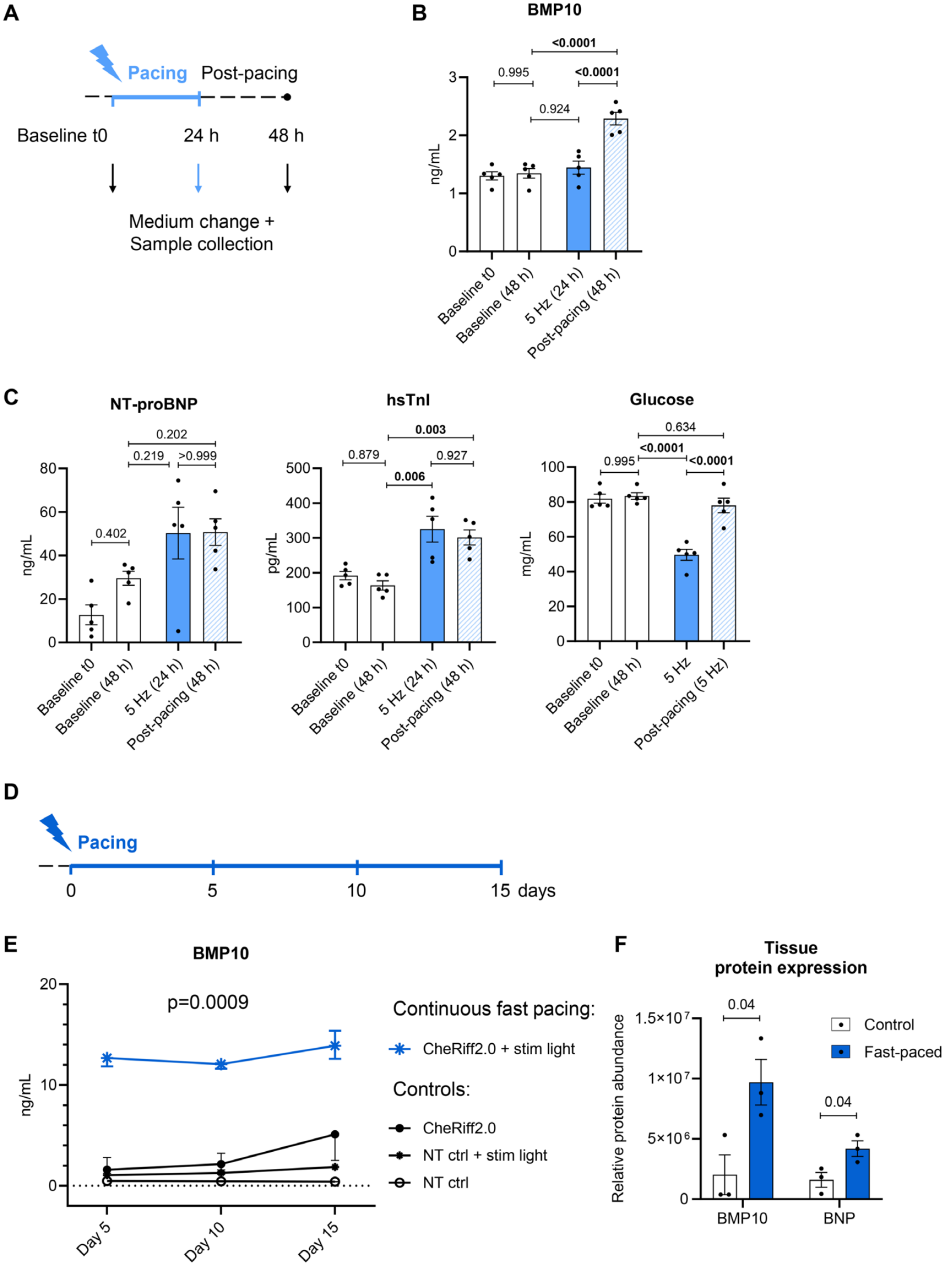
**Media and tissue BMP10 (bone morphogenetic protein 10) content after continuous optogenetic fast pacing. A**, Atrial engineered heart tissues (EHTs) paced at 5 Hz for 24 h or left unpaced (**B** and **C**; n=5/1). First medium sample taken directly after pacing, second sample 24 h after the end of pacing (post-pacing) or 24 h after unpaced control culture. Quantification of (**B**) BMP10 and (**C**) hsTnI (high-sensitivity troponin I) to assess cell damage, NT-proBNP (N-terminal pro-B-type natriuretic peptide) to mark cardiomyocyte stress, and glucose to reflect higher glucose consumption in fast-beating EHTs. Data show glucose concentrations after 24 h of culture. Baseline t0 refers to the time point just before pacing, Baseline (48h) refers to the unpaced, time-matched control. One-way ANOVA followed by Šidák multiple comparisons test. Adjusted *P* values are reported. **D**, Atrial EHTs continuously optically paced 15 days at 4.5 Hz (**E** and **F**). **E**, BMP10 concentration in aEHT medium (accumulated during 48 h) throughout the protocol. Paired *t* test, fast-paced vs controls. **F**, Relative BMP10 and BNP protein abundance in aEHTs, analyzed by mass spectrometry-based proteomics after 15 days of continuous fast pacing. Student *t* test, n=3/group. Contractility data, see Figure S2. Ctrl indicates control; NT, non-transduced; and t0, time point 0 at the start of the protocol.

We paced aEHTs for 24 hours at 5 Hz, followed by a post-pacing period of 24 hours each, enabling time-course analysis of BMP10 secretion (Figure 2A). Optogenetic fast pacing for 24 hours did not immediately elevate BMP10 medium concentrations. The increase in BMP10 by up to 1.7-fold was delayed to 24 hours after pacing during the post-pacing period (Figure 2B). Neither onset nor extent of BMP10 release showed rate dependence across 3, 4, and 5 Hz of applied fast pacing (Figure S2). Pacing of aEHT led to an immediate increase in the release of NT-proBNP and troponin I within 24 hours (Figure 2C). Fast pacing led to significantly increased glucose consumption (Figure 2C). ANP release was highly variable and possibly more volatile (Figure S2 and S3). Effects were similar across 3, 4, and 5 Hz of applied fast pacing (Figure S2).

Continuous optogenetic pacing for up to 15 days (Figure 2D) led to consistently elevated BMP10 release from aEHT. After 5 days of continuous fast pacing at 4.5 Hz, BMP10 release was increased by ≈6-fold, without further increase until day 15 (Figure 2E). Long-term fast pacing caused increased protein expression of both BMP10 and BNP (Figure 2F).

### BMP10 Plasma Concentrations Are Higher in Patients With Current AF

**Figure 3.**
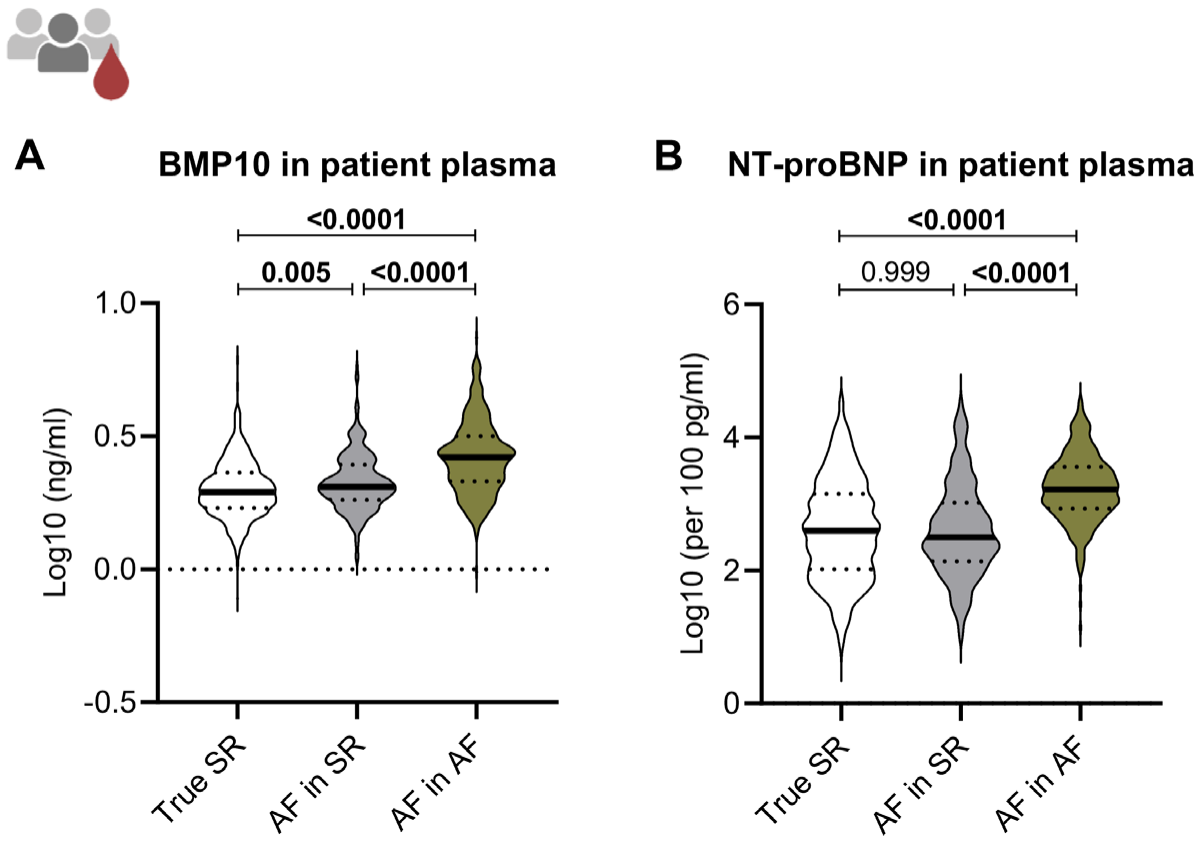
**BMP10 (bone morphogenetic protein 10) and NT-proBNP (N-terminal pro-B-type natriuretic peptide) plasma concentrations in patients by rhythm.** Log10-transformed (**A**) BMP10 and (**B**) NT-proBNP plasma concentrations by rhythm at blood draw. BMP10 was quantified in the plasma of patients without atrial fibrillation (AF; true sinus rhythm [SR]), with diagnosed atrial AF but in SR on the day of blood draw (AF in SR), and patients with AF in AF on the day of blood draw. n=True SR: 814, AF in SR: 254, AF in AF: 302. Median and quartiles depicted. One-way ANOVA followed by Tukey multiple comparisons test, adjusted *P* values reported for all comparisons.

Plasma BMP10 concentrations differed between patients depending on their rhythm status. Patients without AF and therefore in true sinus rhythm, both at blood draw and during the consecutive week, had the lowest BMP10 plasma concentrations, followed by those with a history of AF or Holter ECG-diagnosed AF within 7 days after the blood draw, but in sinus rhythm at the time of blood draw. Highest BMP10 plasma concentrations were observed in patients with AF on the day of blood draw (Figure 3A). In contrast, NT-proBNP concentrations did not differ between sinus rhythm patients and patients with AF in sinus rhythm at blood draw (Figure 3B).

### Atrial EHT and vEHT Express BMP10 Receptors

**Figure 4.**
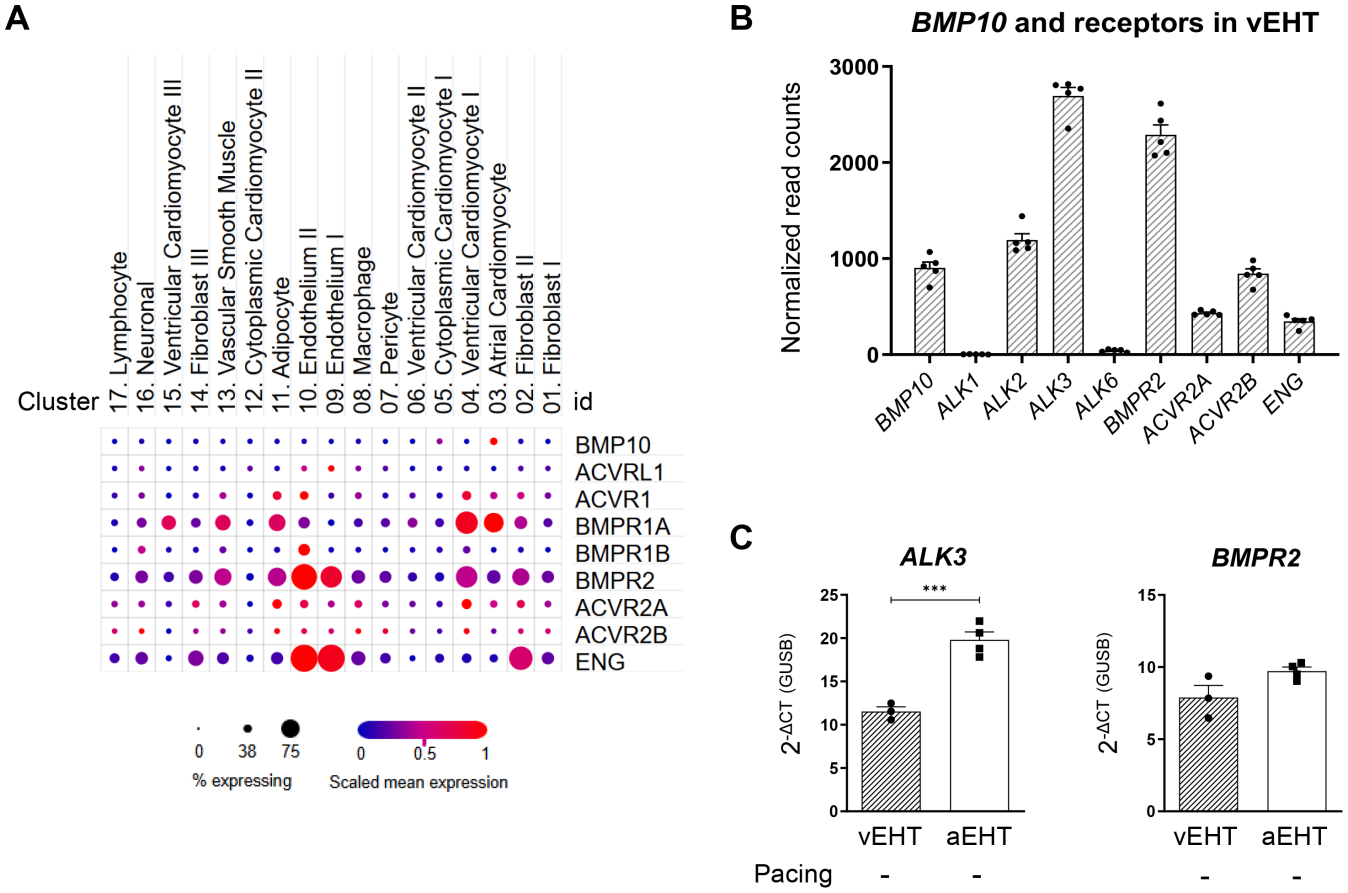
**Expression of BMP10 (bone morphogenetic protein 10) and its receptors in the human heart, ventricular engineered heart tissue (vEHT) and atrial engineered heart tissue (aEHT). A**, Expression of BMP10 and its known receptors in different cell populations of the adult human heart derived from single nuclei RNA sequencing analyses,^20^ via https://singlecell.broadinstitute.org/single_cell. **B**, BMP10 receptor expression in vEHT (bulk RNA sequencing, n=5/1). **C**, Relative mRNA abundance of *ALK3 (BMPR1A*) and *BMPR2* in vEHT and aEHT, quantitative polymerase chain reaction, n=3 to 4/1. Unpaired *t* test; ***P<0.001. *ACVRL*, activin A receptor like type; *ACVR*, activin A receptor type; *BMPR1A*, bone morphogenetic protein receptor type 1A; bone morphogenetic protein receptor type 2; *ENG*, endoglin.

Apart from endothelial cells and fibroblasts, both ventricular and atrial cardiomyocytes of the human adult heart express known BMP receptors (Figure 4A). We identified *ALK3 (BMPR1A*) and *BMPR2* as the BMP receptors with the highest expression in vEHT (Figure 4B) and confirmed expression of both in aEHT (Figure 4C).

### BMP10 Induces Gene Expression Changes Related to AF, Driven by TGFβ Signaling

**Figure 5.**
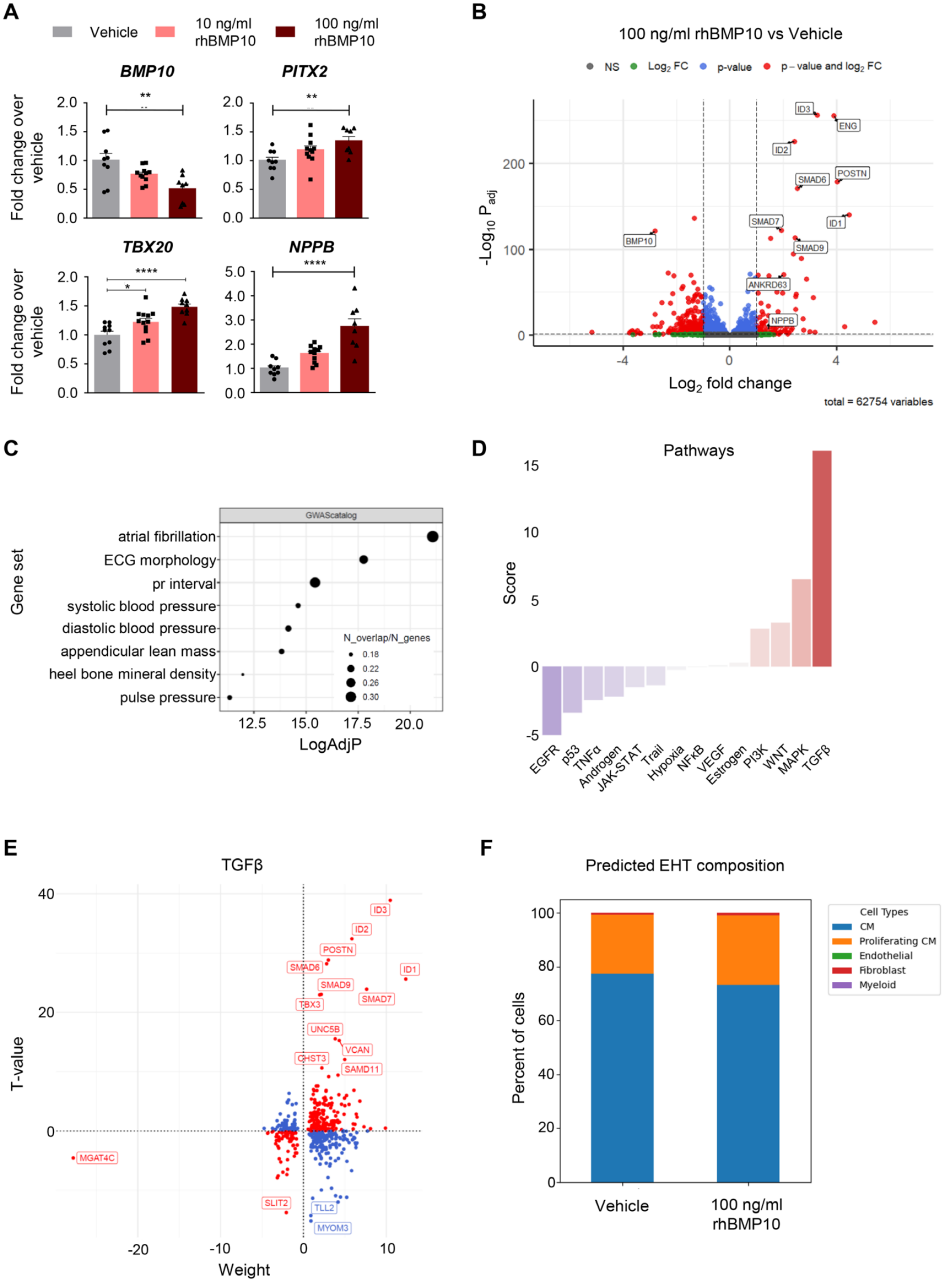
**BMP10 (bone morphogenetic protein 10)-induced gene expression shifts in ventricular engineered heart tissue (vEHT). A**, Gene expression of vEHT as assessed by reverse transcriptase quantitative polymerase chain reaction after 31 to 38 days of culture and 10 days of rhBMP10 (recombinant human BMP10) exposure at 10 ng/mL or 100 ng/mL rhBMP10. 2^−^Δ^CT^ normalized to *GUSB* and vehicle group (n=8–12/2). One-way ANOVA followed by Bonferroni multiple comparisons test,*P<0.05, **P<0.01, ****P<0.0001. **B**, RNA sequencing of 100 ng/mL rhBMP10-exposed vs vehicle control vEHT (n=5/1. **C**, Significantly enriched gene sets. **D**, Pathway analysis and (**E**) underlying expression changes in TGF (transforming growth factor)-β signaling transcripts. Red indicates the matching directionality of expression change in pathway-annotated transcripts. **F**, Cell type content of vEHT predicted by deconvolution analysis of RNA sequencing data. Individual engineered heart tissue (EHT) data can be found in the Supplemental Material. CM indicates cardiomyocyte; FC, fold change; and NS, not significant.

*BMP10* expression in vEHT was low at baseline, but we still observed a significant decrease in expression in response to rhBMP10 exposure. Other cardiac genes linked to atrial function and upregulated by rhBMP10 in a concentration-dependent fashion included *PITX2*, *NPPB*, and *TBX20* (Figure 5A). Expression of the transcription factor *NKX2-5* was not affected by rhBMP10 exposure at the concentrations investigated.

These changes in expression of key atrial genes by chronic rhBMP10 exposure were confirmed in RNA sequencing analysis (Figure S4). Additionally, the strongest expression increase induced by exposure to rhBMP10 was observed for ID genes (*ID1*, *ID2*, *ID3*), SMADs (*SMAD6*, *SMAD9*), and endoglin (*ENG*), a BMP10 receptor-encoding gene (Figure 5B; Figure S5). Gene ontology analysis suggested the terms AF, ECG morphology, and PR interval as the strongest hits (Figure 5C). Pathway analysis revealed the highest score for the TGFβ pathway, driven by ID genes, SMADs, and *POSTN* (Figure 5D and 5E). Deconvolution analysis indicated that rhBMP10 did not affect cell type composition of EHT (Figure 5F; Figure S6). Gross differences in EHT morphology were also excluded (Figure S7).

### BMP10 Exposure Affects Contraction Time of vEHT

Exposing vEHT acutely to increasing concentrations of rhBMP10 or vehicle for 30 minutes each did not affect contractility (Figure S8).

**Figure 6.**
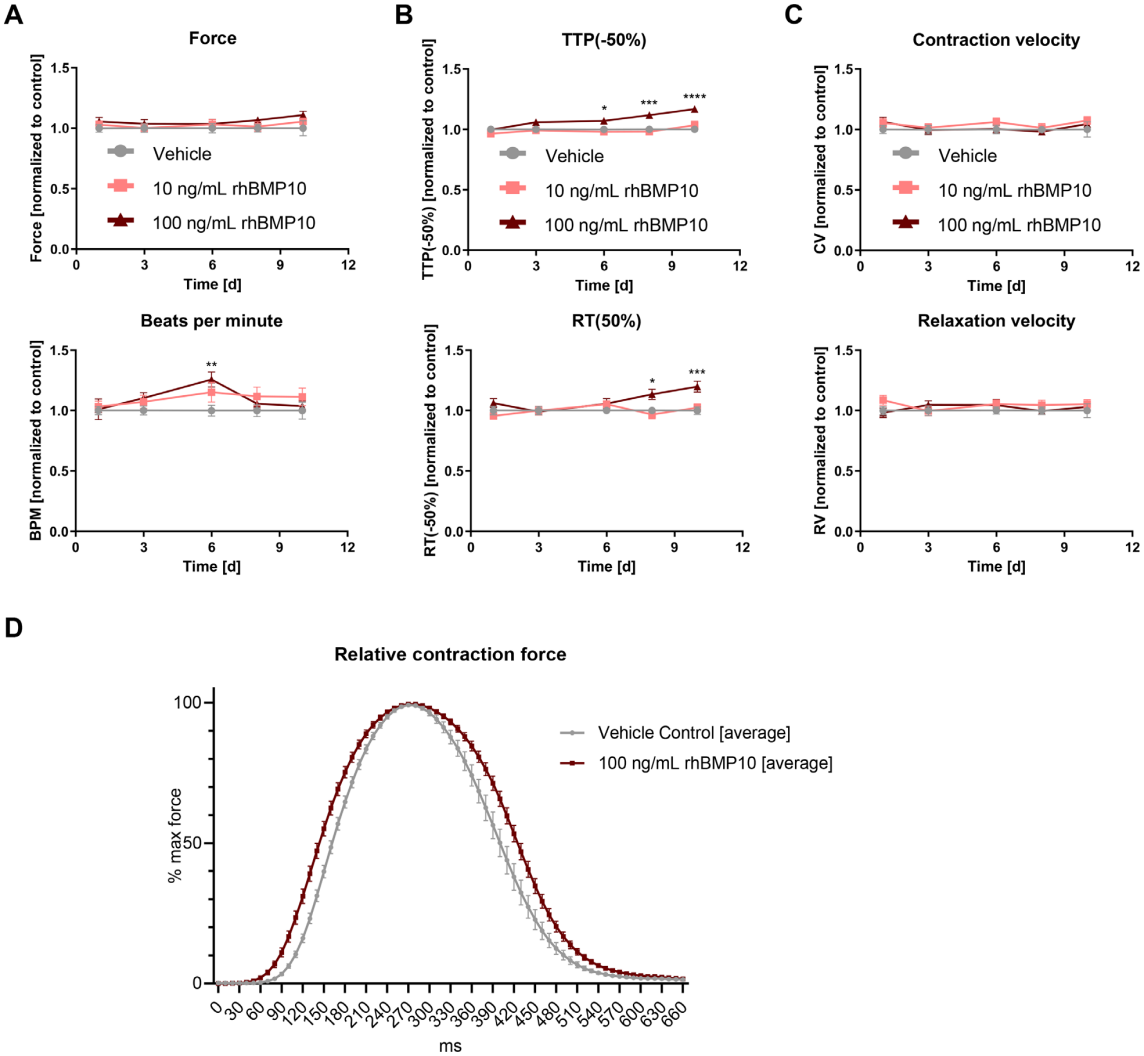
**Contractility development of ventricular engineered heart tissue (vEHT) during longer-term exposure to rhBMP10 (recombinant human BMP10 [bone morphogenetic protein 10]).** Ventricular EHTs were exposed to 10 ng/mL rhBMP10 or to 100 ng/mL rhBMP10 for 10 days (n=14–19/2). **A**, Force development and beats per minute (BPM). **B**, Time to peak (TTP; −50%) and relaxation time (RT; 50%), and (**C**) contraction (CV) and relaxation velocity (RV) are shown. TTP (−50%) refers to the time from 50% to 100% maximal force. All values are normalized to those of the vehicle group of the respective batch. Two-way ANOVA followed by Šidák multiple comparisons test, adjusted*P<0.05, **P<0.01, ***P<0.001, ****P<0.0001. **D**, Averaged, normalized contraction peaks on day 10. Plotted are the normalized mean contraction force as a function of time, aligned by peak.

Longer-term exposure to rhBMP10 for 10 days resulted in increased time to peak (TTP −10%, −50%, and −80%; Figure 6B; Figure S9) as well as increased relaxation time (RT 10%, 50%, and 80%; Figure 6B; Figure S9), without affecting basal force (Figure 6A) or contraction and relaxation velocity (Figure 6C). Averaged, normalized contraction peaks depict the rhBMP10-induced widening of the peak compared with control (Figure 6D).

## Discussion

This analysis of BMP10 release patterns in human atrial and ventricular engineered heart tissue (aEHT/vEHT) and BMP10 in patient plasma yielded the following main findings:

1. BMP10 is expressed and secreted by aEHT.
2. High pacing rates increase BMP10 expression as well as release in aEHT, and aEHT-released BMP10 is biologically active on cardiac fibroblasts.
3. BMP10 plasma concentrations are most elevated in patients with current AF during blood draw. BMP10 concentrations are higher in patients with intermittent AF, but in sinus rhythm at blood draw, than in patients in true sinus rhythm.
4. Ventricular EHT expresses BMP receptors, and exposure to BMP10 leads to TGFβ-related gene expression associated with AF and heart failure.
5. BMP10 prolongs relative contraction and relaxation times in vEHT.

### High Atrial Rates Increase BMP10 Release From aEHT

Consistent with the higher BMP10 secretion in aEHTs with higher pacing rates and higher BMP in patients with AF, a decrease of BMP10 and NT-proBNP concentrations was seen in patients after restoration of sinus rhythm for 3 months.^21,22^

Optogenetic pacing of aEHT allowed us to study prolonged fast excitation at rates comparable to atrial rates in patients with AF without induction of pacing-related toxicity. Fast pacing increased metabolic demand, as evidenced by elevated glucose consumption, and triggered a stress response, reflected by increased BNP expression and release, as well as higher troponin concentration in the media. Fast pacing of aEHT also increased BMP10 and NT-proBNP expression and secretion, aligning with the atrial biomarker profiles observed in AF patients.^10^ Notably, troponin and BMP10 were not released concurrently, arguing against the possibility that BMP10 increase in the media was merely a result of cell death and rupture. Ranging in the low nanograms per millilitre concentrations, the BMP10 concentrations detected in the aEHT media were comparable to those found in patient blood. In patients with AF, both BMP10 and NT-proBNP (encoded by *NPPB*) plasma concentrations are elevated, and this increase is associated with recurrent AF.^6,8,10,22,23^

The delayed release of BMP10 suggests a possible counter-regulatory function of BMP10, for example, in maintaining cardiomyocyte metabolism,^24^ proliferation,^25^ or protection against cell death. The increased BMP10 release appeared slower than that of NT-proBNP and did not correlate with ANP release patterns, arguing for either different release mechanisms of BMP10 and natriuretic peptides altogether or a delayed BMP10 loading of vesicles, if released via the same route. The delayed but continuous BMP10 release after the tissue experienced high rates may correspond with higher BMP10 plasma concentrations in patients with intermittent paroxysmal AF, but in sinus rhythm during blood draw.

### BMP10 Impacts Gene Expression and Contraction of vEHT

Ventricular EHT provides a suitable model to explore the effects of BMP10 exposure due to the low to undetectable intrinsic BMP10 expression, hence serving as an intrinsic control unaffected by varying endogenous BMP10 concentrations. This is important, as BMP10 concentrations will differ across cardiac regions. For instance, in a canine model, tachypacing at 600 bpm for 7 days reduced BMP10 expression in the left atrium (right atrium not studied).^26^

Consistent with prolonged BMP10 exposure upregulating *NPPB*, a key gene expressed in ventricular hypertrophy and heart failure, elevated BMP10 concentrations have also been found in heart failure,^27^ probably including patients with concomitant AF and heart failure.

Similar changes in gene expression and function, like the observed progressive delay in contraction and relaxation, resulting in broadening of the contraction peak shoulders in the vEHT chronically exposed to BMP10, have been reported during the development of tachycardiomyopathy. ^28,29^

### Clinical Perspective

This study provides robust evidence that high atrial rates lead to delayed and sustained release of BMP10 at concentrations that are sufficient to explain a 30% to 100% elevation of BMP10 plasma concentrations. These findings provide a translational explanation for the association of BMP10 with AF found in patients.^6,8,10,27^ The information on the timeline of BMP10 elevation during and after high-rate pacing will allow a more precise interpretation of BMP10 plasma concentrations measured in patients, potentially enabling refinement of AF prediction.^22,30^

The study also identifies a subtle but robust effect of recombinant BMP10 on the contractile function of ventricular engineered heart tissue and induced signaling associated with AF. The results provide a potential mechanism explaining the association of elevated BMP10 concentrations with heart failure events in patients.^11,27,30^ Pending confirmation in other models and in patients, these functional results may suggest that BMP10 could be a marker for the development of arrhythmia-induced cardiomyopathy, a condition that is probably underdiagnosed in clinical care.^31^

### Limitations

Strengths of this study include the evaluation of BMP10 secretion and of effects of recombinant BMP10 on cardiac contractile function in the controlled human EHT model. This allowed direct cause-and-effect attributions. The controlled model is also the main limitation of the study. Although EHT offers a powerful and controllable model for studying cardiac biomarker dynamics and the effects of electrical stimulation, it does not fully recapitulate the cellular complexity, architecture, and electrophysiological properties of the native human heart, and the results should ideally be confirmed by investigation of mammal hearts and human heart tissue. The pacing frequencies studied here (3–5 Hz) induce AF-related remodeling in aEHT within 1 week.^32^ Nonetheless, other aspects contributing to atrial remodeling, such as atrial dilation, irregular activation on repolarization, and effects of the autonomic nervous system, are not fully replicated in the aEHT model. Although the effects of recombinant BMP10 on the contractile function of vEHTs support a role of BMP10 for remodeling, other factors that are secreted in response to high-rate pacing may contribute to atrial remodeling in aEHTs. The study primarily focuses on cardiomyocytes; however, BMP receptors are also expressed in other cardiac cell types, including endothelial cells and fibroblasts. The contribution of these nonmyocyte populations to BMP10 signaling and downstream effects remains unclear, and their role requires further investigation. Additionally, the effects of BMP10 compete with other effects, such as oxidative and metabolic stress, both in this model and in patients. Further research into the effects of BMP10 on cardiac function and its potential contribution to atrial cardiomyopathy, to arrhythmia-induced cardiomyopathy, and to the vicious twins of AF and heart failure is warranted.

### Conclusions

High atrial rates increase BMP10 expression and release, and BMP10 blood concentrations are higher in patients with current AF than in patients with AF in sinus rhythm. BMP10 released by atrial EHT activates BMP signaling in cardiac fibroblasts, and high BMP10 concentrations induce AF- and heart failure-related transcript networks in ventricular EHT. These findings support a role of BMP10 as a biomarker for AF and identify BMP10 as a potential player in AF-induced remodeling and tachycardiomyopathy.

## Article Information

### Acknowledgments

The authors thank Dr Ingke Braren, UKE Vector facility, for expert virus cloning and production, and the Core Facility Genomics of the Medical Faculty of the University of Muenster, for RNA library preparation and sequencing.

### Sources of Funding

This study was cofunded by UKE starter grants to Drs Fabritz and Kirchhof, by UKE Close-the-gap grant to Dr Sommerfeld, by EU 633196 [CATCH ME] to Drs Fabritz and Kirchhof, EU 965286 [MAESTRIA] to Dr Fabritz, British Heart Foundation Accelerator Award (AA/18/2/34218) to University of Birmingham, Deutsches Zentrum für Herz-Kreislauf-Forschung (DZHK, German Center for Cardiovascular Research) supported by the German Ministry of Education and Research, including postdoc start-up grant to Dr Sommerfeld; German Research Foundation (Ki 509167694, STE2596/4-1/456060636, STE2596/5-1/528380599, INST 337/15-1, INST 337/16-1, INST 152/837-1, and INST 152/947-1 FUGG). Dr O’Shea received a Sir Henry Wellcome Fellowship 221650/Z/20/Z.

### Disclosures

Dr Kirchhof received research support from the European Union (EU), British Heart Foundation (BHF), Leducq Foundation, Medical Research Council (MRC) (UK), Else-Kröner-Fresenius-Stiftung, Dutch Heart Foundation, and Deutsches Zentrum für Herz-Kreislauf-Forschung (DZHK, German Center for Cardiovascular Research), from several drug and device companies active in atrial fibrillation (AF), and has received honoraria from several such companies in the past, but not in the last 3 years. Drs Kirchhof and Fabritz are listed as inventors on patents held by the employing institution (AF therapy WO 2015140571, markers for AF WO 2016012783). Dr Fabritz received institutional research grants from the EU, BHF, DZHK, MRC (UK), National Institute for Health and Care Research (NIHR), and several biomedical companies active in the field of research. BMP10 (bone morphogenetic protein 10) and NT-proBNP (N-terminal pro-B-type natriuretic peptide) in patient blood were quantified by Roche as an in-kind contribution as partners of the EU Horizon 2020 CATCH-ME consortium. The other authors report no conflicts.

### Supplemental Material

Supplemental Methods

Tables S1–S2

Figures S1–S9

References 33–40

## Supplementary Material


