# Supplementary material for: High Rate Triggers Increased Atrial Release of BMP10, A Biomarker for Atrial Fibrillation and Stroke, and BMP10 Affects Ventricular Cardiomyocytes
Source: Circ Arrhythm Electrophysiol. 2025 Oct 15;18(11):e013834. doi: 10.1161/CIRCEP.125.013834 (PMC12629124; doi:10.1161/CIRCEP.125.013834)
Supplement: Supplementary file 1 [file hae-18-e013834-s001.pdf]

Supplemental Material to

High rate triggers increased atrial release of BMP10, a biomarker for atrial fibrillation and stroke, and BMP10 affects ventricular cardiomyocytes

Laura C. Sommerfeld, PhD[1,2,3,*]; Jessica Schrapers[3,4,*]; Karl-Felix Müller, BSc[3,4]; Laura Bravo Merodio, PhD[5,6]; Bente Siebels, MSc[7]; A. M. Stella Vermeer-Stoter, PhD[3,4]; Bangfen Pan, PhD[3,4]; Grit Höppner[4]; Christopher O'Shea, PhD[6,8]; Julius Ridder, MSc[1,2,3]; Hartwig Wieboldt[1,2]; Paulina Sander, PhD[1,3]; Tanja Zeller, PhD[1,3]; Winnie Chua, PhD[6]; Yanish J. V. Purmah, MD[6,9]; Robert S. Gardner, PhD[10]; Nathan R. Tucker, PhD[10]; Paulus Kirchhof, MD[2,3,6]; Marc N. Hirt, MD, PhD[2,3,4]; Thomas Eschenhagen, MD[3,4]; Justus Stenzig, MD, PhD[3,4,#]; Larissa Fabritz, MD[1,2,3,6,#]

[1]University Center of Cardiovascular Science UCCS, University Medical Center Hamburg-Eppendorf UKE, Martinistr. 52, 20246 Hamburg, Germany

[2]Department of Cardiology, University Heart and Vascular Center Hamburg, Germany

[3]DZHK (German Center for Cardiovascular Research), partner site Hamburg/Kiel/Lübeck, Germany

[4]Institute of Experimental Pharmacology and Toxicology, UKE, Hamburg, Germany

[5]Cancer and Genomic Sciences, University of Birmingham B15 2TT, United Kingdom

[6]Cardiovascular Sciences, University of Birmingham B15 2TT, United Kingdom

[7]Center of Diagnostics, Section Mass Spectrometry and Proteomics / Core Facility Mass Spectrometric Proteomics, UKE, Martinistr. 52, 20246 Hamburg, Germany

[8]Division of Biomedical Sciences, Warwick Medical School, Clinical Sciences Research Laboratory, CV4 7AL Coventry, UK

[9]Sandwell and West Birmingham Hospitals NHS Trust, B66 2QT Smethwick, UK

[10]SUNY Upstate Medical University, Department of Pharmacology, 766 Irving Avenue

Syracuse, NY 13210

[*,#]These authors contributed equally

Supplemental Methods

EHT generation, culture and contractility analysis (aEHT/vEHT)

Both atrial and standard, ventricular cardiomyocytes (aCM/vCM) were differentiated from established human induced pluripotent stem cell lines of healthy donors. All cell lines employed in this work have been published (UKEi001-A, UKEi003-C), detailed information is available on https://hpscreg.eu. Cardiac differentiation was carried out following published protocols for both, aCM[13] and vCM[15]. Fibrin-based EHT in 24-well cell culture format was created from $1x10^6$ aCM/vCM per EHT. After the onset of spontaneous beating activity at approximately 5-10 days after casting, contractility (force, frequency, rhythmicity, contraction and relaxation velocity) was analyzed every 2-3 days, 1 h after culture media change utilizing pattern recognition software (EHT Technologies and Consulting Team Machine Vision, CTMV, Pforzheim, Germany) following established protocols.[13,15]

Short-term electrical pacing for contractility analysis was carried out with custom-manufactured carbon electrodes (EHT Technologies).[33,14] EHTs were paced at 1.25-2.25 Hz with biphasic impulses (4 ms, 2.25-4 V/cm, Grass S88X stimulator, Natus Neurology Incorporated). Pacing stimulus frequency and electrical field strength were titrated for each batch and set just above pacing threshold and spontaneous beating frequency. Averaged contraction peaks across treatment conditions were visualized for comparison by peak alignment of normalized traces, and the mean contraction as a function of time calculated.

Optogenetic fast pacing of aEHT

A custom-manufactured LED-bearing circuit board (designed and manufactured by Julius Hansen, Sarcura GmbH, Klosterneuburg, Austria; circuit print by JLCPCB, Hong Kong, China) was placed underneath the 24-well cell culture plate harboring the EHTs.

To enable longer-term optogenetic pacing (3-5 Hz), the light-sensitive non-selective cation channel CheRiff2.0 was employed. Atrial EHTs were transduced during casting with an adeno-associated virus vector (AAV6) conferring channel expression under the control of a cardiomyocyte-specific cTNT promoter. LEDs were addressed to rhythmically elicit blue light pulses (465 nm, 0.12 mW/mm2, 45 ms stimuli) by an Arduino controller (Nano ATMega328) using Arduino IDE software.

For control conditions, aEHTs were left to beat spontaneously at intrinsic beating frequency (usually 2 to 2.5 Hz).

Differentiation of hiPSCs to quiescent cardiac fibroblasts (hiPSC-CF)

Differentiation of hiPSCs to quiescent cardiac fibroblasts (hiPSC-CF) was performed according to an adapted version of a previously published protocol (Zhang et al., 2019). Variation of the published protocol included maintenance of hiPSCs in FTDA medium rather than E8 essential medium and coating of cell culture vessels with Geltrex rather than Matrigel. Human iPSCs were seeded on 6-well plates and mesoderm induction was initiated at 70-80% confluency by Wnt activation with 6 µM CHIR99021 (Cayman) in RPMI 1640 medium supplemented with B27. Consequently, cardiac progenitor cell differentiation was initiated by

exposure to 5 µM of the Wnt inhibitor IWR-1 (Selleckchem) in RPMI 1640 supplemented with B27. Cells were then further differentiated to proepicardial cells. After dissociation with Accutase and replating at low density (1.5 million cells/T75), cells were maintained in Advanced DMEM/F12, containing 1% glutamine, 5 µM CHIR99021, 2 µM retinoic acid (Sigma-Aldrich), 5 µM Y-27632 and 1% heat-inactivated FBS for 1 day. Y-27632 and FBS were then removed from the culture medium and 2 days later, CHIR99021 and retinoic acid were also removed. Another 2 days later, cells were dissociated with Accutase and again plated at low density (1.5 million cells/T75). 2 µM SB-431542 were added to the culture medium. After the proepicardial stage, cardiac fibroblast differentiation was induced. Cells were first dissociated with Accutase and plated at low density (1.5 million cells/T75) after which they were maintained in Fibroblast Growth Medium 3 (PromoCell), containing 20 ng/ml bFGF and 10 µM SB-431542. The medium was refreshed every second day. Once the cells were confluent, they were again detached with Accutase and replated at low density (1.5 million cells/T75). After reaching confluency again, CFs could were harvested with Accutase. Cardiac fibroblast differentiation quality control was carried out by morphology and activation assay. This was carried out by re-plating in low serum (0.5%) DMEM and exposure to either 5 µM of the TGFβ receptor inhibitor SB-431542 (quiescent) or to 10 ng/ml TGF-β1 (activated) for at least 24 h, after 24 h of culture. Gene expression was subsequently analysed by RT-qPCR and only fibroblasts with an at least 5-fold difference of *COL1A1* and *POSTN* expression as activation markers between the conditions were used for subsequent experiments.

Fibroblast exposure to conditioned culture media of aEHT

To assess biological activity of aEHT-secreted BMP10, culture media from aEHT were transferred to isogenic hiPSC-derived quiescent cardiac fibroblasts, a cell type known to express BMP10 receptors. Quiescent cardiac fibroblast were exposed to conditioned media collected from i) unpaced aEHT, ii) fast-paced aEHT (4 Hz, 18 days), iii) 10 ng/mL rhBMP10, or iv) vehicle control for 24 h. Media were left on aEHT for 48 h before transfer. To prevent activation of fibroblasts by horse serum contained in the aEHT culture medium, the TGFβ inhibitor
SB-431542 was used (5 µM). Following exposure, fibroblast lysates were probed for SMAD1/5/9 phosphorylation (directly reflecting BMP10 receptor activation) by Western blot.

Protein analysis by Western blot

For protein analysis of EHT after culture, Western blot was employed. EHTs were mechanically disrupted in mPER buffer (Thermo Fisher, Rockford, USA) supplemented with PhosSTOP phosphatase inhibitor (Roche, Mannheim, Germany). Lysates were separated on 10% acrylamide gels, transferred onto nitrocellulose membranes and incubated with the respective primary antibody overnight. Secondary antibody incubation was performed for 2 h before visualization.

BMP10 exposure of vEHT

The relevant abundance of BMP10 receptor transcripts in vEHT suggested that BMP10 could elicit effects on human myocardium and EHT. We therefore exposed vEHT to recombinant human BMP10 protein (rhBMP10) for 10 days.

Effects of BMP10 on ventricular EHT (vEHT) contractility were assessed by both acute (30 min) and longer-term (10 days) exposure of vEHT to recombinant human BMP10 (rhBMP10, 2926-BP-025, R&D Systems, Minneapolis, USA) dissolved in water containing 0.1% bovine serum albumin and 4 mM HCl (vehicle).

For acute experiments, vEHTs were exposed to accumulating concentrations of rhBMP10 (0.5 ng/mL, 2.0 ng/mL, 10 ng/mL, 25 ng/mL, 100 ng/mL and 250 ng/mL) for 30 min each. Contractility was analyzed. To allow for detection of potential positive inotropic effects, in contrast to standard EHT culture employing 1.8 mM extracellular Ca^{2+}, extracellular Ca^{2+} was reduced to 1 mM.

Gene expression analysis

RNA was extracted from either 31-38 day-old vEHT, or 35 day-old aEHT or 2 day-old quiescent hiPSC-derived cardiac fibroblasts with TRIzol reagent (Invitrogen, Carlsbad, USA). For RT-qPCR, reverse transcription was carried out with the High-Capacity cDNA Reverse Transcription kit (Applied Biosystems, Thermo Fisher, Vilnius, Lithuania) and qPCR using HOT FIREPol EvaGreen qPCR Mix Plus (Solis Biodyne, Tartu, Estonia). Primer sequences can be found in Supplementary Table S2.

RIN >9.2 was confirmed for all samples on an Agilent Tapestation device. Library preparation was carried out with the NEBNext Ultra II Directional RNA kit (New England Biolabs) and libraries were sequenced in single read mode (Nextseq 2000, Illumina), 72 cycles.

Mass spectrometry-based proteomic analysis

For protein quantification by mass spectrometry, EHTs were dissolved and disrupted, the lysate was heat-denatured and sonicated. Proteins were reduced, alkylated and washed and bound to magnetic beads. Quantification was carried out by LC-MS/MS (Vanquish neo UHPLC system, Thermo Fisher) using two-buffer chromatography and online desalting, followed by detection on a quadrupole-orbitrap hybrid mass spectrometer (Exploris 480, Thermo Fisher). Protein mapping and quantification was performed with Proteome Discoverer's CHIMERYS DIA algorithm (v3.1.0.638, Thermo Fisher Scientific) against a reviewed human Swissprot database using Inferys 3.0 fragmentation as prediction model, considering peptides between 7 and 30 amino acids.

Protein digestion into tryptic peptides: EHTs were dissolved in 200 µL 100 mM triethyl ammonium bicarbonate and 1% w/v sodium deoxycholate buffer and processed in a bead mill followed by boiling at 95 °C for 5 min and sonication with a probe sonicator. The protein concentration of denatured proteins was determined by the Pierce bicinchoninic acid assay (BCA) protein assay kit (Thermo Fisher) and samples were diluted to 20 µg of protein in 50 µL buffer. Disulfide bonds were reduced in 10 mM dithiothreitol for 30 min at 56 °C and alkylated in presence of 20 mM iodoacetamide for 30 min at 37 °C in the dark. Then, the samples were

dissolved to a concentration of 70% acetonitrile (ACN) and 2 µL carboxylate modified magnetic beads (Sera-Mag Speedbead carboxylate-modified [E3] and magnetic [E7] particles, Cytiva, Marlborough, USA) at 1:1 ratio in LC-MS grade water were added following the single-pot, solid-phase enhanced sample preparation (SP3)-protocol workflow.[34] Samples were shaken at 1400 rpm for 18 min at room temperature. Tubes were placed on a magnetic rack and the supernatant was removed. Magnetic beads were washed two times with 100% ACN and two times with 70% ethanol on the magnetic rack. After resuspension in 50 mM ammonium bicarbonate, digestion with trypsin was performed (sequencing grade, Promega) at 1:100 (enzyme:protein) ratio at 37 °C overnight while shaking at 1400 rpm. Tryptic peptides were bound to the beads by adding 95% ACN and shaken at 1400 rpm for 10 min at room temperature. Tubes were placed on the magnetic rack, the supernatant was removed, and the beads were washed two times with 100% ACN. Elution was performed with 2% DMSO in 1% formic acid. The supernatant was dried in a vacuum centrifuge and stored at -20 °C until further use.

LC-MS/MS data acquisition in DIA mode: Peptides were diluted in 0.1 % formic acid (FA) and 1 µg was injected to the LC-MS/MS system. Chromatographic separation of peptides was achieved with a two-buffer system (buffer A: 0.1% FA in H_2O, buffer B: 0.1% FA in ACN) on a UHPLC (Vanquish neo UHPLC system, Thermo Fisher). Attached to the UHPLC was a peptide trap (300 µm x 5 mm, C18, Trap Cartridge, Thermo Fisher) for online desalting and purification, followed by a 25 cm C18 reversed-phase column (75 µm x 250 mm, 130 Å pore size, 1.7 µm particle size, peptide BEH C18, nanoEase, Waters). Peptides were separated using an 80 min method with linearly increasing ACN concentration from 2% to 30% ACN over 60 minutes.

MS/MS measurements were performed on a quadrupole-orbitrap hybrid mass spectrometer (Exploris 480, Thermo Fisher). Eluting peptides were ionized using a nano-electrospray ionization source (nano-ESI) with a spray voltage of 1,800 and analyzed in data independent acquisition (DIA) mode. For each MS1 scan, ions were accumulated for a maximum of 240 ms or until a charge density of 3 x 106 ions (AGC Target) was reached. Fourier-transformation based mass analysis of the data from the orbitrap mass analyzer was performed covering a mass range of m/z 400 – 1,400 with a resolution of 120,000 at m/z = 200. Within a precursor mass range of m/z 380-980 fragmentation in DIA-mode with m/z 12 isolation windows and m/z 1 window overlaps was performed. Fragmentation was performed at normalized collision energy of 28% using higher energy collisional dissociation (HCD). An AGC target of 2 x 106 ions or a maximum of 54 ms was set. Orbitrap resolution was set to 30 000 with a scan range from m/z 350-2000.

LC-MS/MS data processing and analysis: LC-MS/MS data were searched with the CHIMERYS DIA algorithm integrated into the Proteome Discoverer software (v3.1.0.638, Thermo Fisher Scientific) against a reviewed human Swissprot database using Inferys 3.0 fragmentation as prediction model. Carbamidomethylation was set as a fixed modification for cysteine residues. The oxidation of methionine was allowed as variable modification. A maximum number of one missing tryptic cleavage was set. Peptides between 7 and 30 amino acids were considered. A strict cut-off (FDR < 0.01) was set for peptide identification. Quantification was performed by CHIMERYS based on fragment ions. Normalization was applied to the total peptide amount. Obtained protein abundances were log2-transformed, and further statistical testing was performed in Perseus (Maxquant, Max-Planck-Institute of

Biochemistry).[35] Student's T-testing was performed and results of p-values and log2 fold changes were visualized as volcano plots in R Studio.

Cell type deconvolution of the bulk RNA sequencing data

The relative proportions of cell types represented in EHT samples were estimated using CIBERSORTx [36,37], a commonly-used reference-based approach to deconvolution. Previously published single cell RNA sequencing data from EHT (Gene Expression Omnibus repository; accession number GSE211650[38]) were used to generate a reference dataset. Available data from all reference samples (i.e., GSM6482038; GSM6482039; GSM6482040) were processed using Scanpy v1.10.3 to determine cell type annotations, as described before[38] with minor modifications. Briefly, cell barcodes were filtered to remove empty droplets. Doublets were identified by Solo[39] and removed from analysis. Cells were further filtered for total counts (≥300 and ≤20000), total genes (≥500 and ≤5500), and counts aligned with mitochondrial genes (≤1%). Data were normalized to total counts, scaled, and log-transformed. PCA was applied to highly variable genes, and coordinates were corrected using Harmony for batch integration. Neighborhood graphs were computed, and Leiden clustering performed to identify relatively unique cell clusters. Wilcoxon statistical tests were performed to identify cell cluster marker genes used to annotate cell types. Cell clusters were visualized in reduced dimensions using the UMAP algorithm. For deconvolution, cells were randomly sampled from the entire dataset, with the maximum number of cells within each cell type set to 1000. Count data scaled to counts per million for each cell type represented in the reference dataset (cardiomyocytes, n=1000; proliferating cardiomyocytes, n=680; fibroblasts, n=1000; endothelial cells, n=575; myeloid cells, n=72) were used as input in CIBERSORTx to generate a signature matrix of cell-type marker genes. Top marker genes (set to 100-200 in CIBERSORTx) were identified as those with an FDR-corrected p-value <0.01, and ranked according to log-fold change across cell types. The signature matrix file was used as reference input in the CIBERSORTx Cell Fractions module with bulk RNA sequencing data scaled to counts per million.

The EHT single cell reference samples used for deconvolution were limited in the types of cells represented, with transcriptomes that may additionally differ from those of cells in cardiac tissue. Therefore, we verified our deconvolution result showing high proportions of cardiomyocytes in our samples using single nuclei ventricular cardiac samples from human donors.[40] Single nucleus RNA count data were processed and annotated as outlined in.[40] Raw count data scaled to counts per million from 200 nuclei in each of cardiomyocytes, fibroblasts, endothelial cells, lymphatic endothelial cells, endocardial cells, macrophages, lymphocytes, mast cells, pericytes, smooth muscle cells, adipocytes, and neuronal cells from COVID-positive and COVID-negative organ donors were used as input to CIBERSORTx to generate a signature matrix. COVID and Non-COVID samples were included to ensure the diversity of cardiac cell activation states were represented in the reference dataset. Parameters for signature matrix generation and cell fraction estimation were as noted above.

Supplemental Tables:

Supplemental Table S1: Clinical characteristics of patients by rhythm

**Characteristic**	**True SR (N=814)**	**AF in SR (N=254)**	**AF in AF (N=302)**
Age, years *	67 (57, 75)	70 (60, 79)	74 (67, 81)
Male sex	483 (59%)	157 (62%)	185 (61%)
Ethnicity			
Caucasian	559 (69%)	213 (84%)	267 (88%)
Asian	160 (20%)	26 (10%)	16 (5%)
Afro-Caribbean	92 (11%)	15 (6%)	19 (6%)
BMI, kg/m^2 *	28.6 (25.1, 32.6)	28.7 (25.0, 32.3)	29.7 (25.2, 34.1)
eGFR, mL/min/1.73 m^2	77.7 (27.7)	77.4 (23.1)	69.9 (27.9)
Diabetes	368 (45%)	51 (20%)	75 (25%)
Stroke/TIA	76 (9%)	27 (11%)	32 (11%)
Coronary artery disease	398 (49%)	63 (25%)	62 (21%)
Arterial hypertension	519 (64%)	128 (50%)	167 (55%)
Heart failure	152 (19%)	36 (14%)	105 (35%)

Categorical variables are reported as n (%), continuous variables are reported as mean (standard deviation) or median (quartile 1, quartile 3) for skewed distributions (*). AF atrial fibrillation, BMI body mass index, eGFR estimated glomerular filtration rate, SR sinus rhythm, TIA transient ischemic attack.

Supplemental Table S2: qPCR Primer sequences

Gene	Primer sequence
BMP10	F: GACATCCCCACGCAGGATTC<br>R: TAAAACTGACCGGCTGGGAA
BMPR1A (ALK3)	F: AGTGCTATTGCTCAGGGCAC<br>R: GGCTTTTGGAGAATCTTTGCACT
BMPR2	F: AATGCAGCCATAAGCGAGGT<br>R: TCTGGTACGGATTCCCCTGG
PITX2	F: AGCCATTCTTGCATAGCTCG<br>R: GTGTGGACCAACCTTACGGA
NPPB	F: TGCAAGGGTCTGGCTGCTTTGG<br>R: CACTTCAAAGGCGGCCACAGGG
TBX20	F: AGTCACTGCCTACCAGAATCAA<br>R: GGCTCTCCACACTTTCCCTCT
NKX2-5	F: CCAAGGACCCTAGAGCCGAA<br>R: CCACCGACACGTCTCACTC
SMAD6	F: CTCCCTACTCTCGGCTGTCT<br>R: TGGCGTCTGAGAATTCACCC
SMAD9	F: CACACAACGCCACCTATCCT<br>R: TGGGGCTCCTCGTAACAAAC
ID1	F: AATCCGAAGTTGGAACCCCC<br>R: CTTCAGCGACACAAGATGCG
ID3	F: GCTTTTGCCACTGACTCGG<br>R: TTTGGTGAAGTCAAGTGGGC

Supplemental Figures and Figure Legends

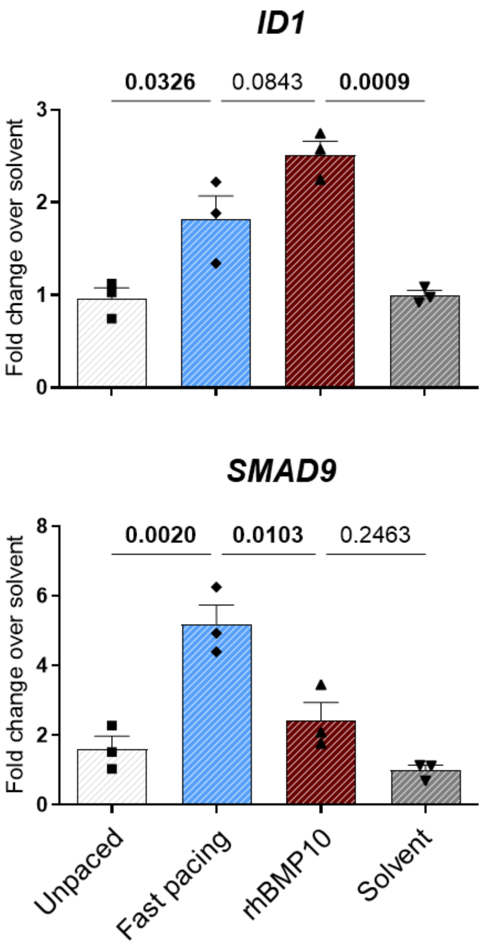

Supplemental Figure S1: qPCR from cardiac fibroblasts confirming higher expression of BMP(10) signaling-specific genes. Human induced pluripotent stem cell-derived quiescent cardiac fibroblasts (hiPSC-CF) were exposed to media transferred from aEHT left at intrinsic beating frequency (unpaced), optogenetically fast-paced at 4 Hz for 18 days, or media containing either 10 ng/mL recombinant human BMP10 (rhBMP10) or its solvent only, for 24 h. Quantitative PCR for BMP10 signaling target genes was carried out. N=3 wells per group. One-way ANOVA followed by Šidák multiple comparisons test. Adjusted *P* values are reported on graphs.

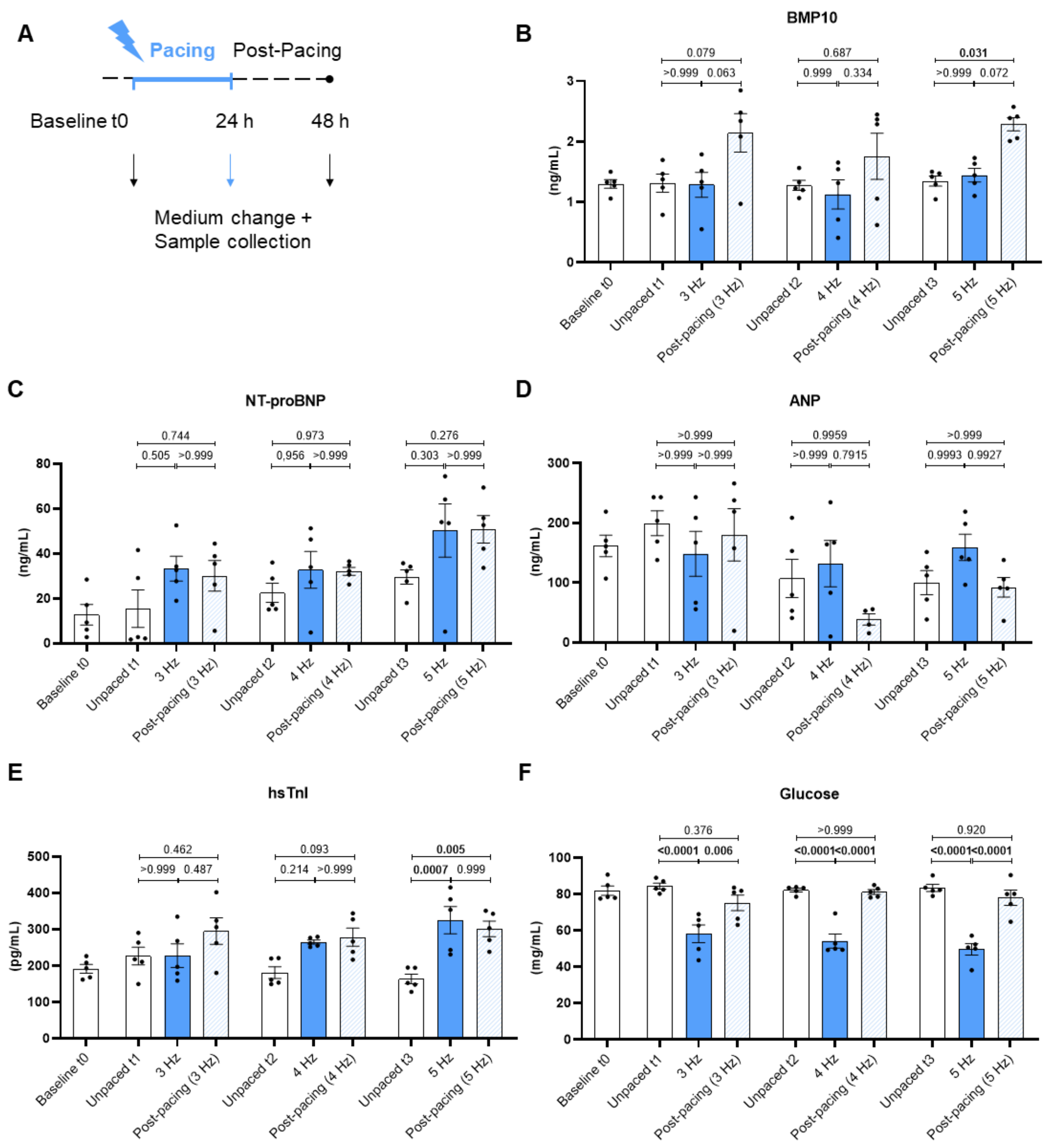

Supplemental Figure S2: Media content of BMP10 and other biomarkers after continuous optogenetic fast pacing. (A) Atrial EHTs were paced at 3, 4 and 5 Hz for 24 h or left unpaced. **(B-F)** Culture medium was assessed by ELISA (n=5/1). First medium sample taken directly after pacing, second sample 24 h after the end of pacing (post-pacing) or 24 h after unpaced control culture. Quantification of **(B)** BMP10, **(C)** N-terminal pro-brain natriuretic peptide (NT-proBNP) as a marker for cardiomyocyte stress, **(D)** atrial natriuretic peptide (ANP), **(E)** troponin I high-sensitivity assay (hsTnI) as a marker for cell damage, and **(F)** glucose for verification of fast pacing by higher glucose consumption in fast-beating aEHTs. One-way ANOVA followed by Šidák multiple comparisons test. Adjusted *P* values are reported on graphs.

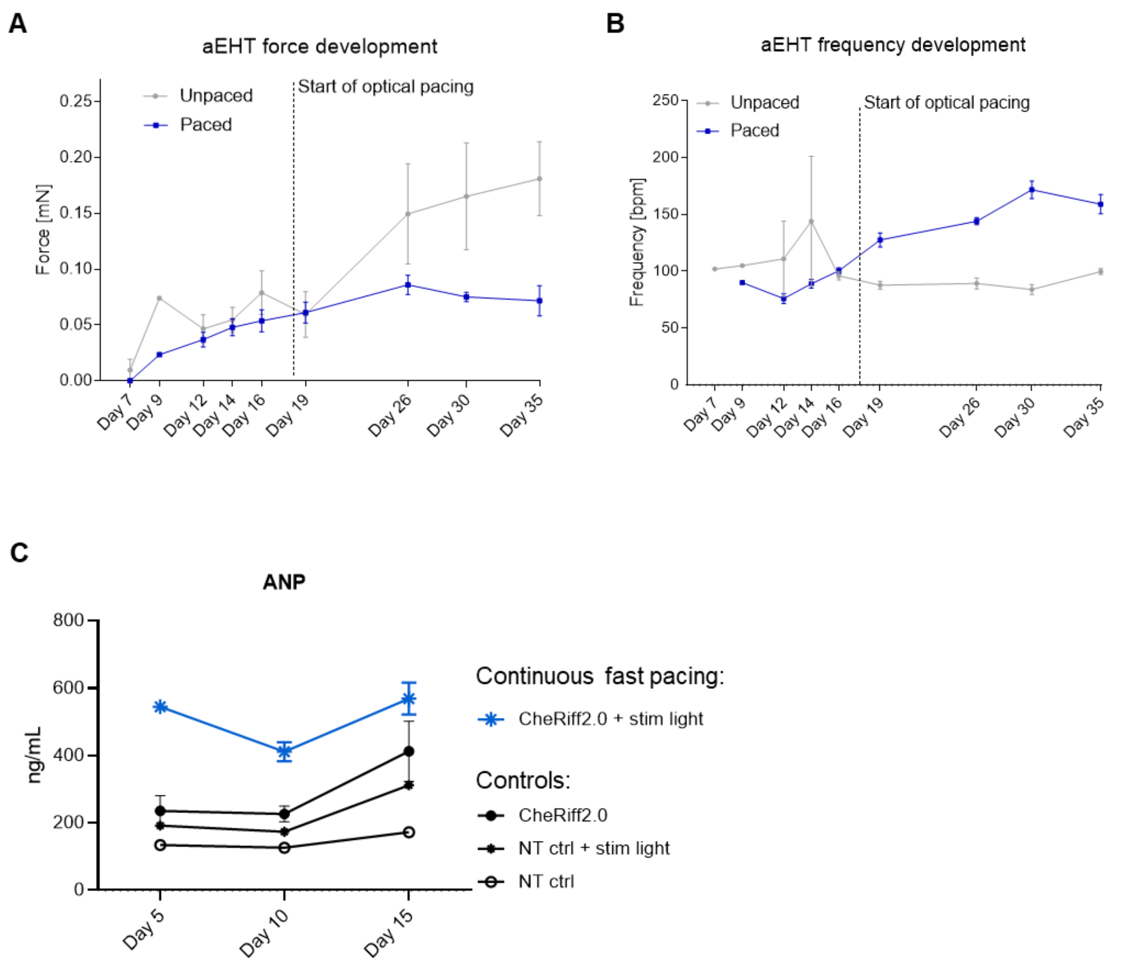

Supplemental Figure S3: Contractility data and ANP media content of aEHT paced for up to 15 days. Development of aEHT **(A)** contraction force and **(B)** intrinsic beating frequency. n=3-4/1. Pacing at 4.5 Hz was continuously applied. Media for BMP10 and ANP release quantification were collected 5, 10 and 15 days after initiation of the pacing protocol (vertical dashed line). **(C)** Atrial natriuretic peptide (ANP) quantified in aEHT media, accumulated within 48 h and collected on the indicated day after pacing initiation, n=2-4/1.

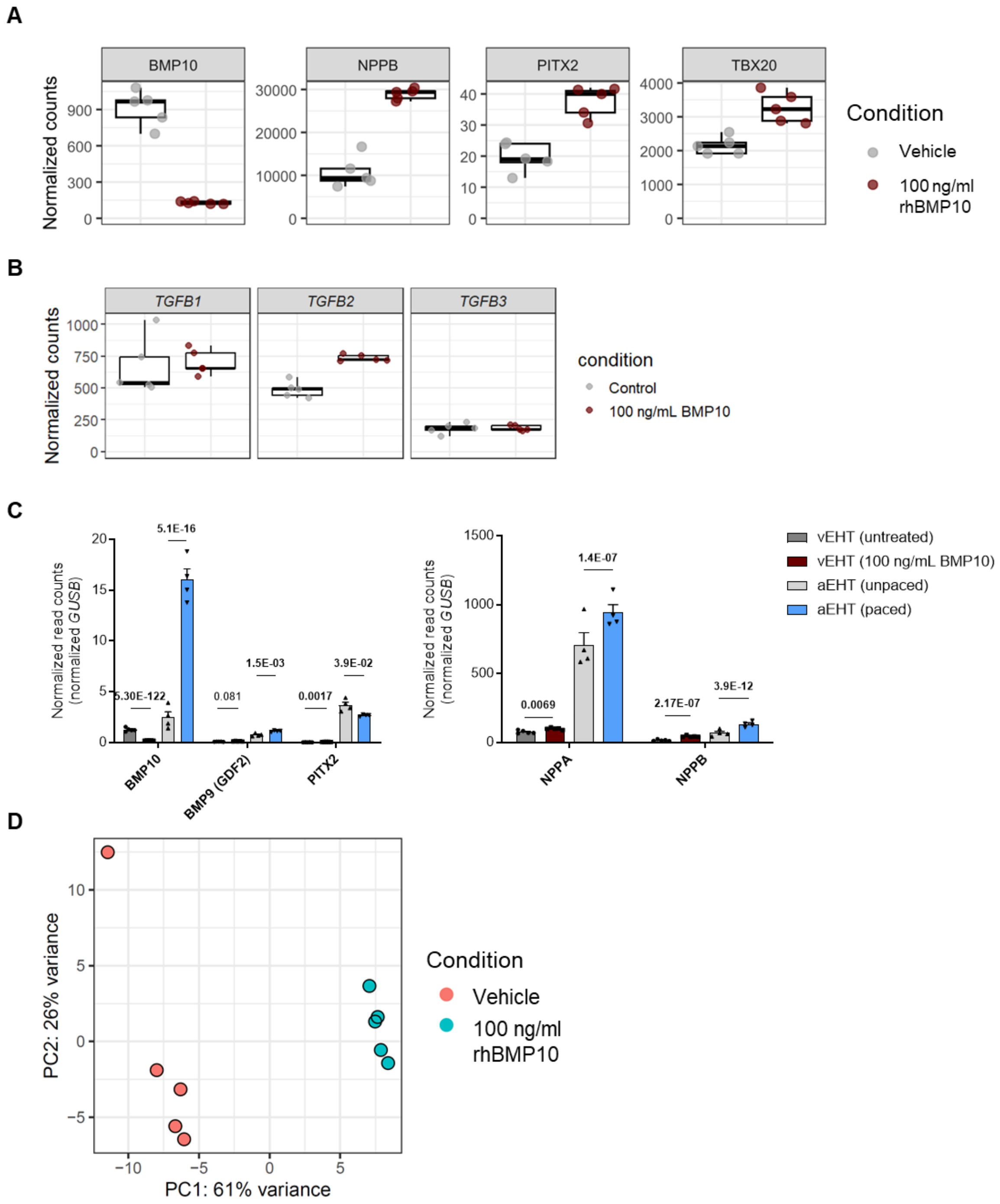

Supplemental Figure S4: RNA sequencing analysis of vEHT after longer-term rhBMP10 exposure. Normalized read counts of **(A)** *BMP10*, *NPPB*, *PITX2* and *TBX20*, as also quantified by qPCR (Figure 4A) and **(B)** TGFβ-encoding genes *TGFB1*, *TGFB2*, *TGFB3*. **(C)** Normalized read counts of *BMP9*, *BMP10*, *PITX2*, *NPPA* and *NPPB* in vEHT reported on in (A) and (B) as well as in aEHT which were left unpaced or fast-paced at 4-5 Hz for 21 days. FDR (P_{adj}) from DESeq2 analyses are reported on graphs. **(D)** PCA plot of RNA sequencing results of vehicle control and rhBMP10 (100 ng/ml)-exposed vEHT, n=5/1.

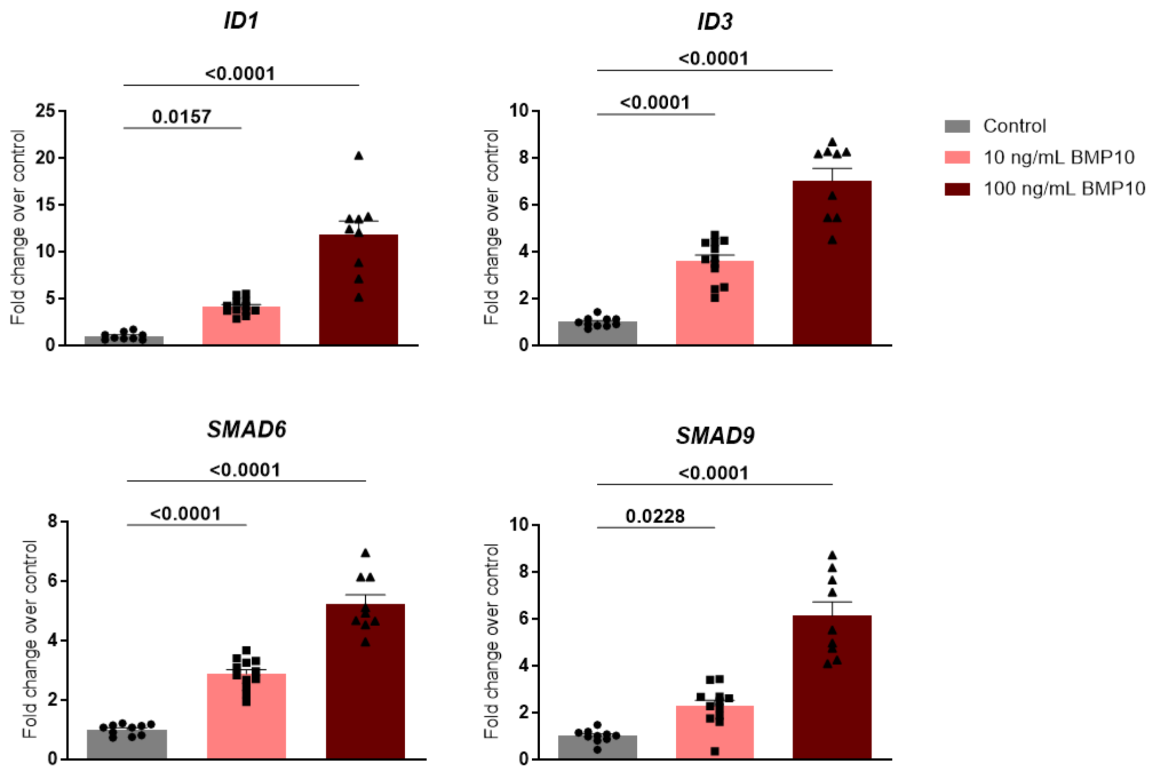

Supplemental Figure S5: Expression of BMP-regulated genes in vEHT. RNA expression assessed by qPCR in vEHT after 10 days of either vehicle (control) or rhBMP10 exposure at 10 ng/mL or 100 ng/mL. N=9-12/2. One-way ANOVA followed by Šidák's multiple comparisons test. Adjusted *P* values are reported on the graphs.

A

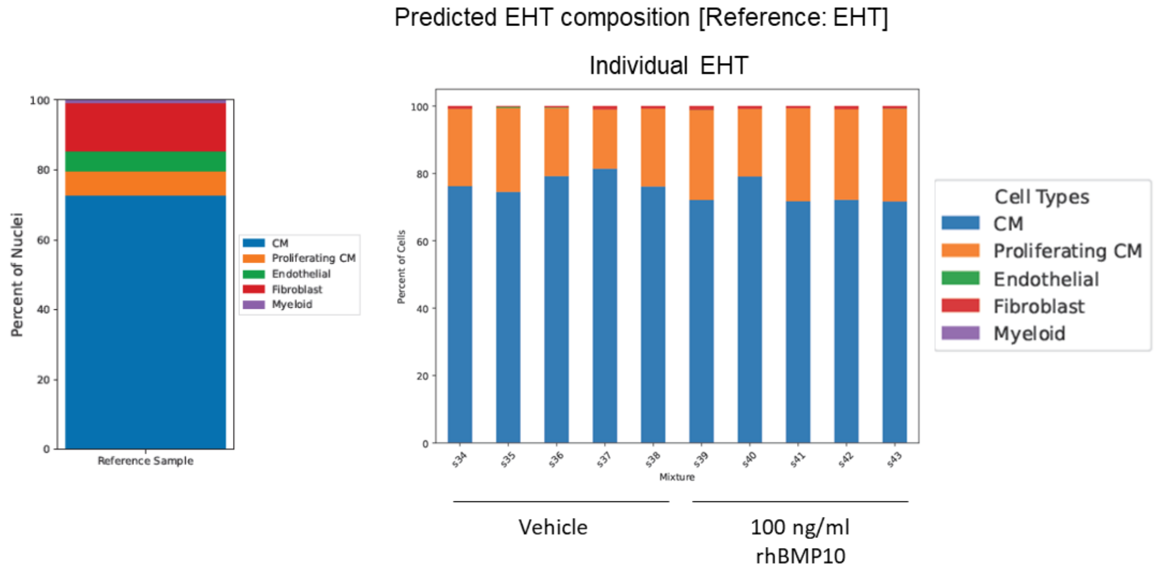

B

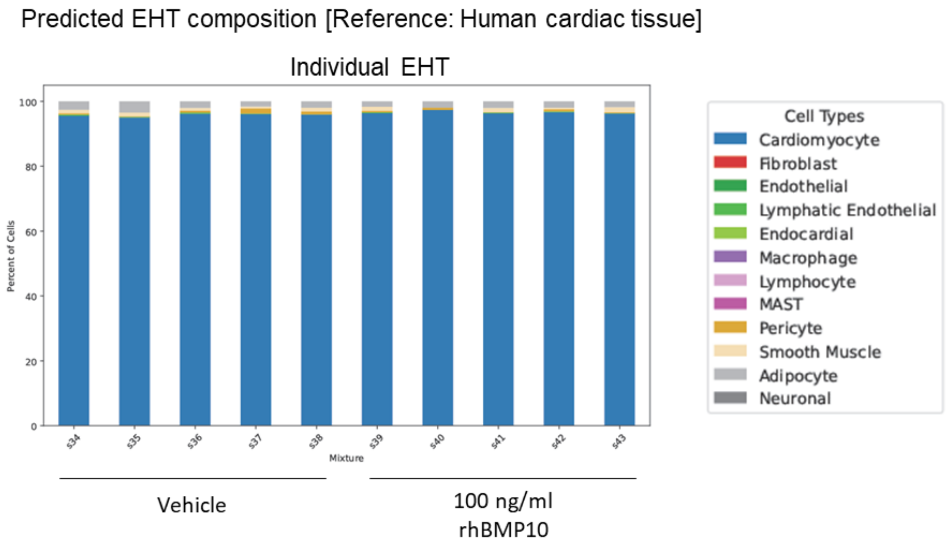

Supplemental Figure S6: Deconvolution of EHT cell composition based on bulk RNA-sequencing results. We inferred cell fractions with CIBERSORTx. **(A)** Reference sample and predicted cell type composition of individual EHT analyzed, using single nuclei sequencing data from EHT.[38] **(B)** Estimated cell type fractions using a human heart COVID single nuclei sequencing data set, including activated fibroblasts.[40] Independent of the reference data sets used, BMP10 exposure did not affect EHT cell type composition.

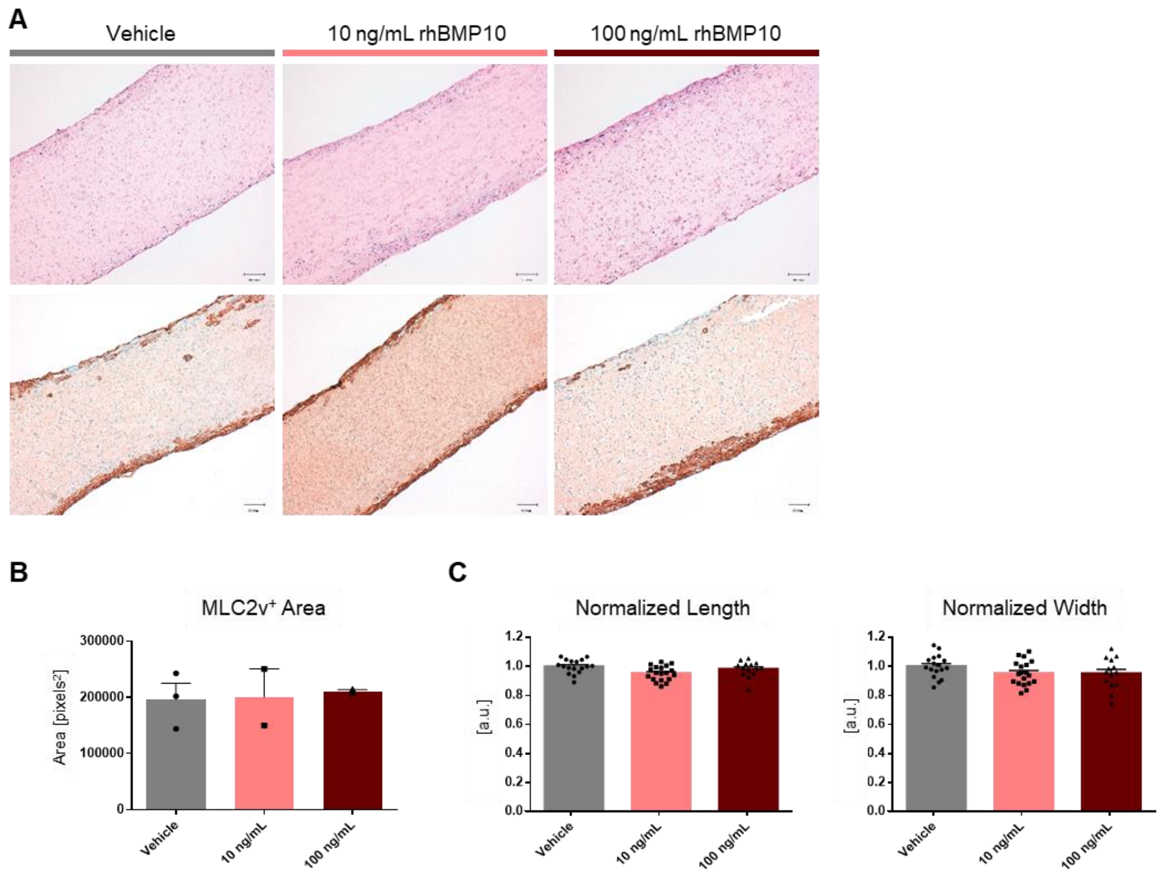

Supplemental Figure S7: Histological appearance of vEHT after longer-term exposure to rhBMP10. (A) H&E staining (upper panel) and MLC2v immunohistochemistry (lower panel) representative microscopy images of vEHT exposed to vehicle (left), 10 ng/mL rhBMP10 (middle) or to 100 ng/mL rhBMP10 for 10 days (right). Scale bar 100 µm. **(B)** MLC2v-positive area, quantified from immunohistochemistry images as in (A, lower panel). **(C)** Diastolic length and width of intact vEHT analyzed from still images of videos from contractility analysis system on last day of culture and normalized to vehicle control.

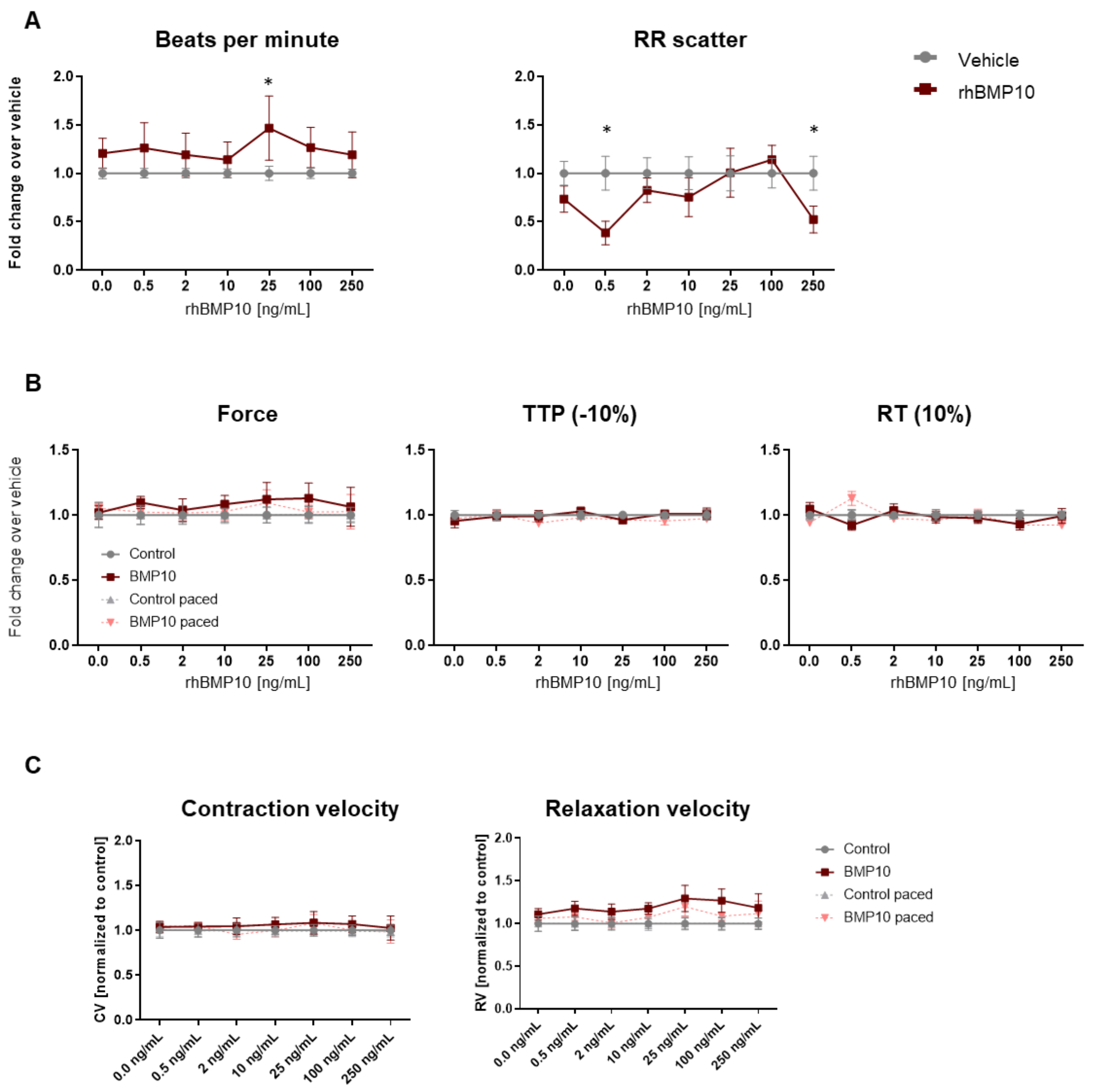

Supplemental Figure S8: Contractility of vEHT during acute exposure to rhBMP10. VEHTs were exposed to increasing concentrations of rhBMP10; 0.5 ng/mL, 2.0 ng/mL, 10 ng/mL, 25 ng/mL, 100 ng/mL, 250 ng/mL; n=7-12/2, for ~30 min each. **(A)** Beats per minute and RR scatter of spontaneously-beating vEHT. Two-way ANOVA followed by Šidák multiple comparisons test. *$P_{adj}<0.05$ **(B)** force development, time to peak (TTP; -10%), relaxation time (RT; 10%) and **(C)** contraction and relaxation velocity are shown. Values normalized to vehicle group of respective batch. Mixed effects analysis, not significant.

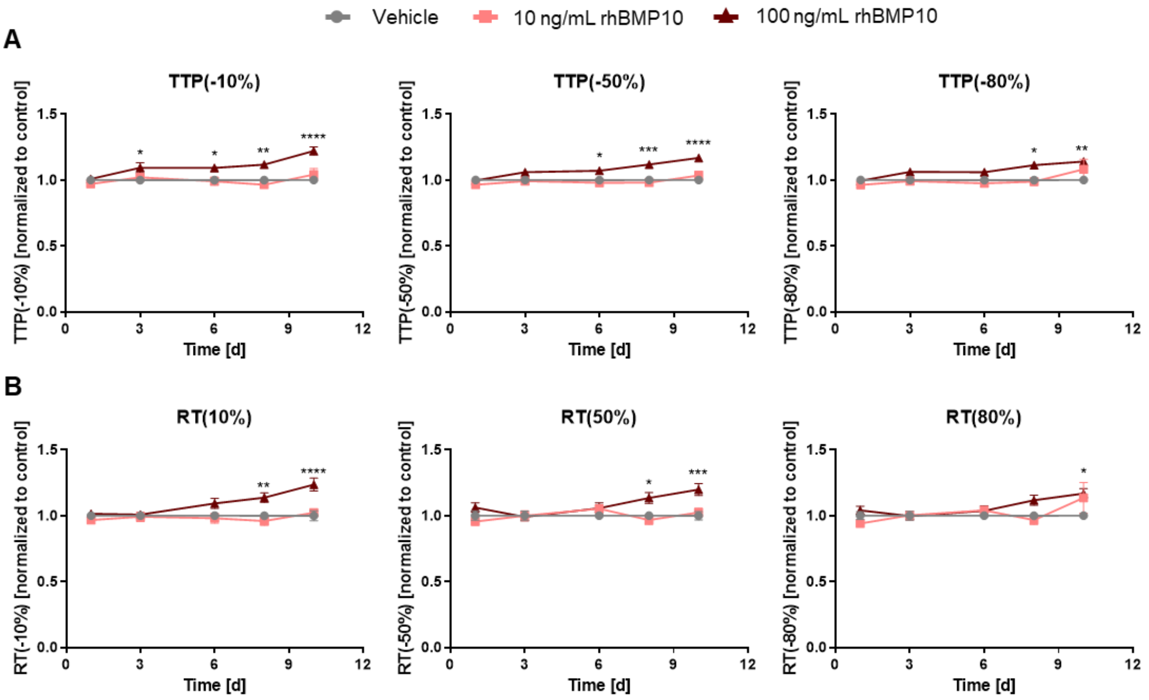

Supplemental Figure S9: Contractility of vEHT during longer-term exposure to rhBMP10. VEHTs were exposed to 10 ng/mL or 100 ng/mL rhBMP10 for 10 days, n=14-19/2. **(A)** Time to peak (TTP; -10%, -50%, -80%) and **(B)** relaxation time (RT; 10%, 50% and 80%). Values normalized to vehicle group of respective batch. Two-way ANOVA followed by Šidák multiple comparisons test. Adjusted **P*<0.05, ***P*<0.01, ****P*<0.001, *****P*<0.0001.

Supplemental References

33. Hirt MN, Boeddinghaus J, Mitchell A, Schaaf S, Bornchen C, Muller C, Schulz H, Hubner N, Stenzig J, Stoehr A, et al. Functional improvement and maturation of rat and human engineered heart tissue by chronic electrical stimulation. J Mol Cell Cardiol. 2014;74:151-161. doi: 10.1016/j.yjmcc.2014.05.009
34. Hughes CS, Moggridge S, Muller T, Sorensen PH, Morin GB, Krijgsveld J. Single-pot, solid-phase-enhanced sample preparation for proteomics experiments. *Nature protocols*. 2019;14:68-85. doi: 10.1038/s41596-018-0082-x
35. Tyanova S, Temu T, Sinitcyn P, Carlson A, Hein MY, Geiger T, Mann M, Cox J. The Perseus computational platform for comprehensive analysis of (prote)omics data. *Nature methods*. 2016;13:731-740. doi: 10.1038/nmeth.3901
36. Newman AM, Liu CL, Green MR, Gentles AJ, Feng W, Xu Y, Hoang CD, Diehn M, Alizadeh AA. Robust enumeration of cell subsets from tissue expression profiles. *Nature methods*. 2015;12:453-457. doi: 10.1038/nmeth.3337
37. Newman AM, Steen CB, Liu CL, Gentles AJ, Chaudhuri AA, Scherer F, Khodadoust MS, Esfahani MS, Luca BA, Steiner D, et al. Determining cell type abundance and expression from bulk tissues with digital cytometry. *Nat Biotechnol*. 2019;37:773-782. doi: 10.1038/s41587-019-0114-2
38. Loos M, Klampe B, Schulze T, Yin X, Theofilatos K, Ulmer BM, Schulz C, Behrens CS, van Bergen TD, Adami E, et al. Human model of primary carnitine deficiency cardiomyopathy reveals ferroptosis as a novel mechanism. *Stem Cell Reports*. 2023;18:2123-2137. doi: 10.1016/j.stemcr.2023.09.002
39. Bernstein NJ, Fong NL, Lam I, Roy MA, Hendrickson DG, Kelley DR. Solo: Doublet Identification in Single-Cell RNA-Seq via Semi-Supervised Deep Learning. *Cell Syst*. 2020;11:95-101 e105. doi: 10.1016/j.cels.2020.05.010
40. Brener MI, Hulke ML, Fukuma N, Golob S, Zilinyi RS, Zhou Z, Tzimas C, Russo I, McGroder C, Pfeiffer RD, et al. Clinico-histopathologic and single-nuclei RNA-sequencing insights into cardiac injury and microthrombi in critical COVID-19. *JCI Insight*. 2022;7. doi: 10.1172/jci.insight.154633
